# The Robustness of Plant-Pollinator Assemblages: Linking Plant Interaction Patterns and Sensitivity to Pollinator Loss

**DOI:** 10.1371/journal.pone.0117243

**Published:** 2015-02-03

**Authors:** Julia Astegiano, François Massol, Mariana Morais Vidal, Pierre-Olivier Cheptou, Paulo R. Guimarães

**Affiliations:** 1 CEFE UMR 5175, CNRS—Université de Montpellier—Université Paul-Valéry Montpellier—EPHE campus CNRS, Montpellier, France; 2 Departamento de Ecologia, Instituto de Biociências, Universidade de São Paulo, São Paulo, Brazil; 3 Laboratoire GEPV, CNRS UMR 8198, Université Lille 1, Villeneuve d’Ascq, France; University of Calgary, CANADA

## Abstract

Most flowering plants depend on pollinators to reproduce. Thus, evaluating the robustness of plant-pollinator assemblages to species loss is a major concern. How species interaction patterns are related to species sensitivity to partner loss may influence the robustness of plant-pollinator assemblages. In plants, both reproductive dependence on pollinators (breeding system) and dispersal ability may modulate plant sensitivity to pollinator loss. For instance, species with strong dependence (e.g. dioecious species) and low dispersal (e.g. seeds dispersed by gravity) may be the most sensitive to pollinator loss. We compared the interaction patterns of plants differing in dependence on pollinators and dispersal ability in a meta-dataset comprising 192 plant species from 13 plant-pollinator networks. In addition, network robustness was compared under different scenarios representing sequences of plant extinctions associated with plant sensitivity to pollinator loss. Species with different dependence on pollinators and dispersal ability showed similar levels of generalization. Although plants with low dispersal ability interacted with more generalized pollinators, low-dispersal plants with strong dependence on pollinators (i.e. the most sensitive to pollinator loss) interacted with more particular sets of pollinators (i.e. shared a low proportion of pollinators with other plants). Only two assemblages showed lower robustness under the scenario considering plant generalization, dependence on pollinators and dispersal ability than under the scenario where extinction sequences only depended on plant generalization (i.e. where higher generalization level was associated with lower probability of extinction). Overall, our results support the idea that species generalization and network topology may be good predictors of assemblage robustness to species loss, independently of plant dispersal ability and breeding system. In contrast, since ecological specialization among partners may increase the probability of disruption of interactions, the fact that the plants most sensitive to pollinator loss interacted with more particular pollinator assemblages suggest that the persistence of these plants and their pollinators might be highly compromised.

## Introduction

The robustness of plant-pollinator assemblages to species loss is a main concern of basic and applied Ecology since 87.5% of flowering plants and most crops directly consumed by humans depend in some degree on pollinators to reproduce [1, 2, 3]. Understanding how the organization of plant-pollinator interactions may influence species persistence is central to predict the consequences of species loss [4]. Ultimately, ecological interactions are shaped by traits and therefore trait-based descriptions of species interactions may provide insights into the organization of interacting assemblages [5]. In this context, a main challenge is to understand how traits shaping species interaction patterns also influence the sensitivity of species to the loss of interaction partners [6, 7].

Plant-pollinator assemblages show consistent patterns in the organization of interactions among species [8]. In these assemblages, only a few species of plants and pollinators are extremely generalized i.e. interact with a high number of species [8]. Generalists are central to the nested structure of plant-pollinator assemblages, in which ecologically specialized species interact with subsets of species interacting with more generalized species [9, 10]. As a consequence, the loss of generalized species decreases the robustness of plant-pollinator assemblages to subsequent species loss [11–13]. Thus, the persistence of generalists may increase the persistence of the whole assemblage [11–13].

In plants, pollination of generalists should be less affected by pollinator loss due to pollinator redundancy [14–18, 19]. Indeed, generalized plants tend to interact both with generalized and specialized pollinators [20], which may lead to lower fluctuation of pollinator service [15] and to lower risk of reproductive failure when specialized pollinators go extinct [21]. However, plant sensitivity to pollinator loss is not only affected by interaction patterns among plants and pollinators but also by plant breeding system [22–24].

Plant breeding systems modulate the dependence of plants on pollinators to produce seeds [22, 24]. Among biotically pollinated plants, self-incompatible and dioecious species are strongly dependent on pollinator services, since pollination will only occur when pollinators have previously visited flowers of compatible and male conspecific plants, respectively. In contrast, self-compatible species may be less dependent on pollinators because a single visit of pollinators to each individual flower may allow reproduction. Plants having mechanisms to produce seeds without pollinator visits may be even less dependent. As generalization on pollinators may evolve if it decreases the risk of reproductive failure when pollinators fluctuate in abundance [15], plants with different breeding systems may show different levels of generalization [25]. Species depending more on pollinators may interact with multiple pollinator species [26] whereas plants interacting with fewer pollinator species may be less dependent on pollinators [27–29]. Moreover, pollinator-dependent plants would be expected to interact with more generalized pollinators, minimizing temporal fluctuation in pollination services [15, 29].

Plant dispersal ability may also modulate plant sensitivity to pollinator loss. Higher dispersal ability has been associated with lower dependence on pollinators (i.e. self-compatibility or autonomous self-pollination) because only plants with the ability to produce seeds without pollinators should reproduce in sites were pollinators are absent (Baker’s law) [30, 31]. In contrast, it has recently been shown in theoretical studies that fluctuations in pollination levels may lead to the evolution of two alternative syndromes: outcrossers with high dispersal ability or selfers with low dispersal [32, 33]. Indeed, high dispersal ability may increase the persistence of dioecious species [34]. In this sense, if strongly dependent species can locally persist by dispersing seeds from sites where pollinators are present, then their persistence may be less dependent on local pollinator services. Thus, the patterns of interaction of plants may also be modulated by dispersal ability, with highly dispersing plants interacting with fewer and/or more specialized pollinator species and low-dispersal plants being more generalists and/or interacting with generalized pollinators.

In this study, we investigated how plant interaction patterns varied with plant sensitivity to pollinator loss. We also explored how this variation might influence the robustness of plant-pollinator assemblages. We compiled information on plant breeding system and dispersal mode for 192 species from 13 published plant-pollinator networks. We described the patterns of interaction for each plant species, i.e. its contribution to network nestedness, its degree of ecological generalization on pollinators [35], and the mean level of generalization of its pollinators. Specifically, we asked how these patterns were associated with the dependence of plants on pollinators and their dispersal ability. We predicted that plants with strong dependence on pollinators or low dispersal ability may show higher degree of generalization on pollinators, higher contribution to nestedness and interact with more generalized pollinators. In addition, we investigated how the association of plant generalization, dependence on pollinators and dispersal ability may affect the robustness of plant-pollinator assemblages by simulating sequences of species extinctions. We expected that scenarios where plant generalization level and biological traits affected the probability of extinction of plants would lead to less robust networks than scenarios only considering plant generalization and as robust as the random scenario—i.e. where no plant trait affected plant sensitivity.

## Methods

### Plant interaction patterns and sensitivity to pollinator loss

We characterized the interaction pattern of 339 plant species belonging to 13 qualitative plant-pollinator networks available at the interaction web database (http://www.nceas.ucsb.edu/interactionweb/; S1 Table). We used three network metrics to characterize the patterns of interaction of each species: plant contribution to nestedness, plant ecological generalization on pollinators (hereafter plant generalization) and mean ecological generalization of plant interaction partners (hereafter mean pollinator generalization). Plant contribution to nestedness measured how much the interactions of a given plant species overlapped, on average, with those of other plant species in the network, following [36] (see “Plant contribution to nestedness” in the supporting information). We first calculated the proportion of pollinator species shared between a given plant and each plant species of the network. Then, for each plant, the average proportion of pollinators shared with the other plant species represented its contribution to nestedness. To calculate plant contribution to nestedness we used the ANINHADO software [37]. The generalization of a given plant species was characterized by the proportion of pollinator species that interacted with it [38], k_p_/S_A_, where k_p_ was the number of pollinator species interacting with the focal plant species and S_A_ was the number of pollinator species of the network. The generalization level of each pollinator interacting with a given plant species was calculated as k_a_/S_P_, where k_a_ was the number of plant species that interacted with a given pollinator and S_P_ was the plant richness species of the network. The mean pollinator generalization for a given plant species was obtained as the mean of the generalization level of all pollinators interacting with the focal plant species.

We used information on breeding system and dispersal mode to estimate, respectively, plant dependence on pollinators and dispersal ability. Data on breeding system and dispersal mode was obtained for 192 of the initial 339 species (58%; S2 Table). For most of the plant species, data on these traits was extracted from published articles (S2 Table). When different studies were available for the same species, we prioritized information obtained in the same study region where networks were characterized. Data on the two traits for 6% of species was obtained from researchers working with those plants. Dispersal mode was also obtained from seed-trait databases (7.5% of species; S2 Table). In 7% of plants, dispersal mode was assigned by the analysis of images of the dispersal units and according to [39].

We grouped the large diversity of plant breeding systems in three categories depicting the degree of dependence of plants on pollinators to produce seeds. Species where classified as: (i) strongly dependent plants, including self-incompatible and dioecious species, and obligate outcrossers; (ii) intermediately dependent plants, including self-compatible species and facultative outcrossers; and (iii) slightly dependent plants, including autonomous self-pollinating, agamospermous, cleistogamous, and facultative autogamous species. Dispersal mode was coded in two classes depicting dispersal ability: (i) low-dispersal plants, including species dispersed by gravity (with or without diaspore explosion), and ants; (ii) high-dispersal plants, including species dispersed by vertebrates, wind and water.

### Statistical analyses

We evaluated how plant interaction patterns varied among plants differing in their dependence on pollinators and dispersal ability. In our analysis, plant generalization, contribution to nestedness and mean generalization of pollinators were the response variables, and dependence on pollinators and dispersal ability the explanatory factors. Since residuals of linear models were not normally distributed but variances among groups were homogeneous, we chose to use the distance-based non-parametric analysis of variance introduced by Anderson [40, 41]. Thus, we computed p-values using permutation tests (n = 9999 permutations). We performed these permutation analyses using the Adonis function included in the Vegan package on R [42]. Particularities of each assemblage such as species richness and connectance (i.e. the proportion of possible interactions actually recorded) could influence the value of the response variables. Thus, permutations were performed among species within networks. To determine *a posteriori* pair-wise differences between levels of factors that influenced the response variables, we performed separated permutation tests following [40]. Data on plants occurring in more than one network (6 species) were included only once in the analyses by randomly choosing one of these networks. Including or not these 6 species in the analyses did not change the results.

### Plant sensitivity to pollinator loss and network robustness

We evaluated how plant sensitivity to pollinator loss might influence network robustness by simulating plant extinctions. We used 10 networks, excluding three networks from this analysis due to limited information on both the dependence of plants on pollinators and plant dispersal abilities (< 60% of the species) (S1 Table). Among these 10 networks, species without information on a given trait were randomly assigned with equal probability to one category of the trait at the beginning of each simulation of extinction events.

We evaluated network robustness under different simulated scenarios of plant extinctions. We used a fully factorial design leading to eight simulated scenarios in which each plant trait (i.e. plant generalization, dependence on pollinators or dispersal ability) or the combination of these traits determined plant sensitivity to pollinator extinction (see S3 Table for a full description of the scenarios). Plant sensitivity depending on species generalization represented the ability of plants to cope with fluctuations on the abundances of pollinators or even pollinator loss, associated with pollinator redundancy (e.g. the reproduction of plants interacting with few pollinators is more likely to be pollen limited [43]). Thus, plants interacting with multiple pollinators were considered to be less sensitive to pollinator loss than those interacting with a few species. In the case of plant dependence on pollinators and dispersal ability, plant sensitivity represented the ability of plants to cope with pollinator decline with alternative strategies, e.g. autonomous self-pollination or seeds arriving from other sites. Then, species with slight dependence on pollinators and/or high dispersal ability (e.g. wind or animal dispersed) were less sensitive to pollinator loss. Simulated scenarios also included a random scenario under which no plant trait affected plant sensitivity. This random scenario represented a null model to compare the effects of the other scenarios on assemblage robustness [11, 13, 44].

To perform extinction sequences, we assigned probabilities of extinction to each plant species according to plant generalization, dependence on pollinators and dispersal ability as follows. For scenarios only considering one of these traits, we first ranked all the species according to a decreasing order of sensitivity to pollinator loss, as explained above. Thus, in the case of plant dependence on pollinators, strongly dependent species were considered the most sensitive whereas slightly dependent species were the least sensitive. In relation to dispersal ability, low-dispersal plants were considered the most sensitive whereas high-dispersal species were the least sensitive. In the case of plant generalization on pollinators, higher generalization was associated with lower sensitivity to pollinator loss. Secondly, for the N species within each category of each trait we sampled N extinction probabilities from a truncated exponential distribution, where the lambda parameter was randomly chosen among values ranging from 10 to 25. Thus, species belonging to more sensitive categories were first ranked and received higher extinction probabilities. Then, we computed the mean extinction probability of the sampled values for each trait category and assigned this mean value to all plants within the category. Finally, we normalized the probability values across all species (i.e. we made the sum of all probabilities equal to 1) to obtain the final probability of extinction for each species. In scenarios taking into account any combination of traits, we assigned extinction probabilities according to each trait as explained above, and we multiplied the set of probabilities for each species. Then, we normalized these probability values across species in order to obtain the final probability of extinction for each species. In the random scenario, all species received the same probability of extinction calculated as 1/S_P_.

We performed 1000 sequences of plant extinction for each scenario. Each sequence included the sequential removal of all plant species. A pollinator species died out if all their interacting plants were extinct. After the removal of each plant species, we computed the percentage of surviving pollinator species. Then, we assessed the robustness of the networks following each sequence of extinction by computing the area under the curve describing the proportion of remaining pollinator species against the proportion of plants that went extinct [45, 46]. Areas that are close to one represent networks that are robust to plant extinction, since large percentages of extinctions are needed until significant secondary extinctions of pollinators are observed. On the other hand, areas that are close to zero correspond to very fragile networks, in which extinction of a small proportion of plant species leads to the extinction of a high proportion of pollinator species [45]. Simulations were performed in MATLAB [47]. We obtained mean values of robustness for each scenario and then we compared the differences between means of a-priori planned pair-wise comparisons (see S5–S12 Tables). We calculated the 95% confidence intervals for differences between means and we considered that the difference between scenarios was significant when the confidence interval did not include the zero value [48].

## Results

### Plant interaction patterns and sensitivity to pollinator loss

**Figure 1:**
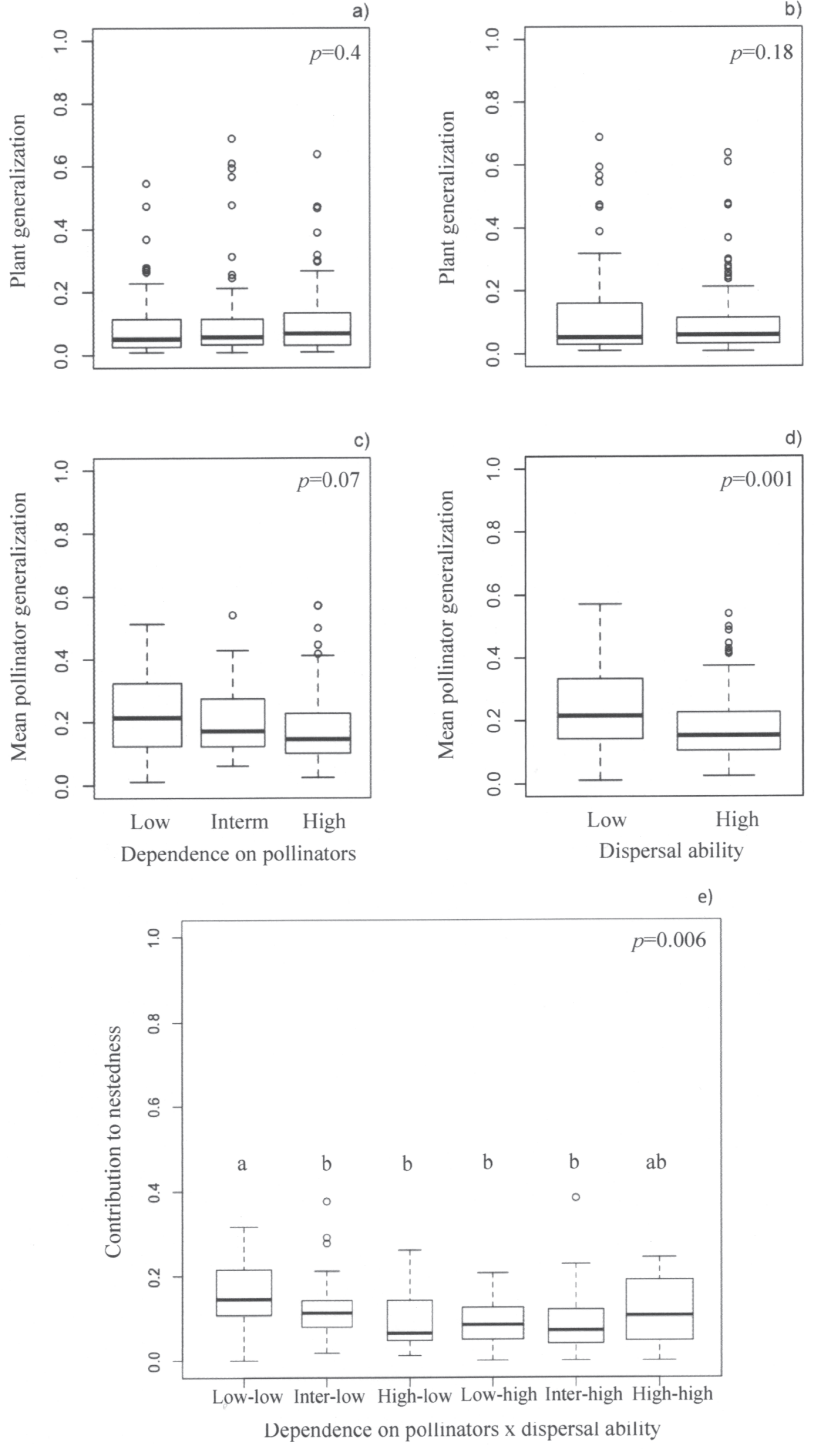
Plant interaction patterns and sensitivity to pollinator loss. Box-plots of plant generalization (“a” and “b”), mean pollinator generalization (“c” and “d”) and plant contribution to nestedness (“e”) of species differing in dependence on pollinators and dispersal ability. Black lines within boxes represent median values. Upper and lower limits of boxes represent 1^st^ and 3^rd^ quartiles, respectively. Boxes were drawn with widths proportional to the number of observations in each group. In (a) and (c), “Strong”, “Inter” and “Slight” refer to strongly, intermediately and slightly dependent plants, respectively. In (b) and (d), “Low” and “High” refer to high and low-dispersal plants, respectively. In (e) “Strong-Low”, “Inter-Low” and “Slight-Low” refer to plants with strong, intermediate and low dependence on pollinators and slight dispersal ability, whereas “Strong-high”, “Inter-high” and ‘Slight-high” refer to plants with high, intermediate and low dependence on pollinators and high dispersal ability. The probability of obtaining a difference higher than that observed among groups is also shown. Abbreviations: Inter = intermediate.

Plant generalization did not differ among species with different dependence on pollinators (*F*_1, 192_ = 0.68, *P* = 0.4; Fig. 1A and B; S4 Table) or dispersal ability (*F*_1, 192_ = 1.85, *P* = 0.18; Fig. 1A and B; S4 Table). However, low-dispersal plants interacted, on average, with more generalized pollinators (*F*_1, 192_ = 11.26, *P* = 0.0013; Fig. 1D; S4 Table). Low-dispersal plants showed higher contribution to nestedness than high-dispersal plants (*F*_1, 192_ = 7.62, *P* = 0.006; Fig. 1E; S4 Table and S1 Fig.). However, the relationship between dispersal ability and nestedness was modulated by plant dependence on pollinators (*F*_1, 192_ = 7.19, *P* = 0.006; Fig. 1E and S4 Table). Thus, species with low dispersal ability and slight dependence on pollinators showed the highest contribution to nestedness (Fig. 1E). Species with strong dependence on pollinators and high dispersal ability showed intermediate mean values of contribution to nestedness (Fig. 1E) that did not differ significantly from the other groups. By contrast, low-dispersal plants with strong dependence on pollinators were among those that showed the lowest contributions to nestedness (Fig. 1E).

### Plant sensitivity to pollinator loss and network robustness

**Figure 2:**
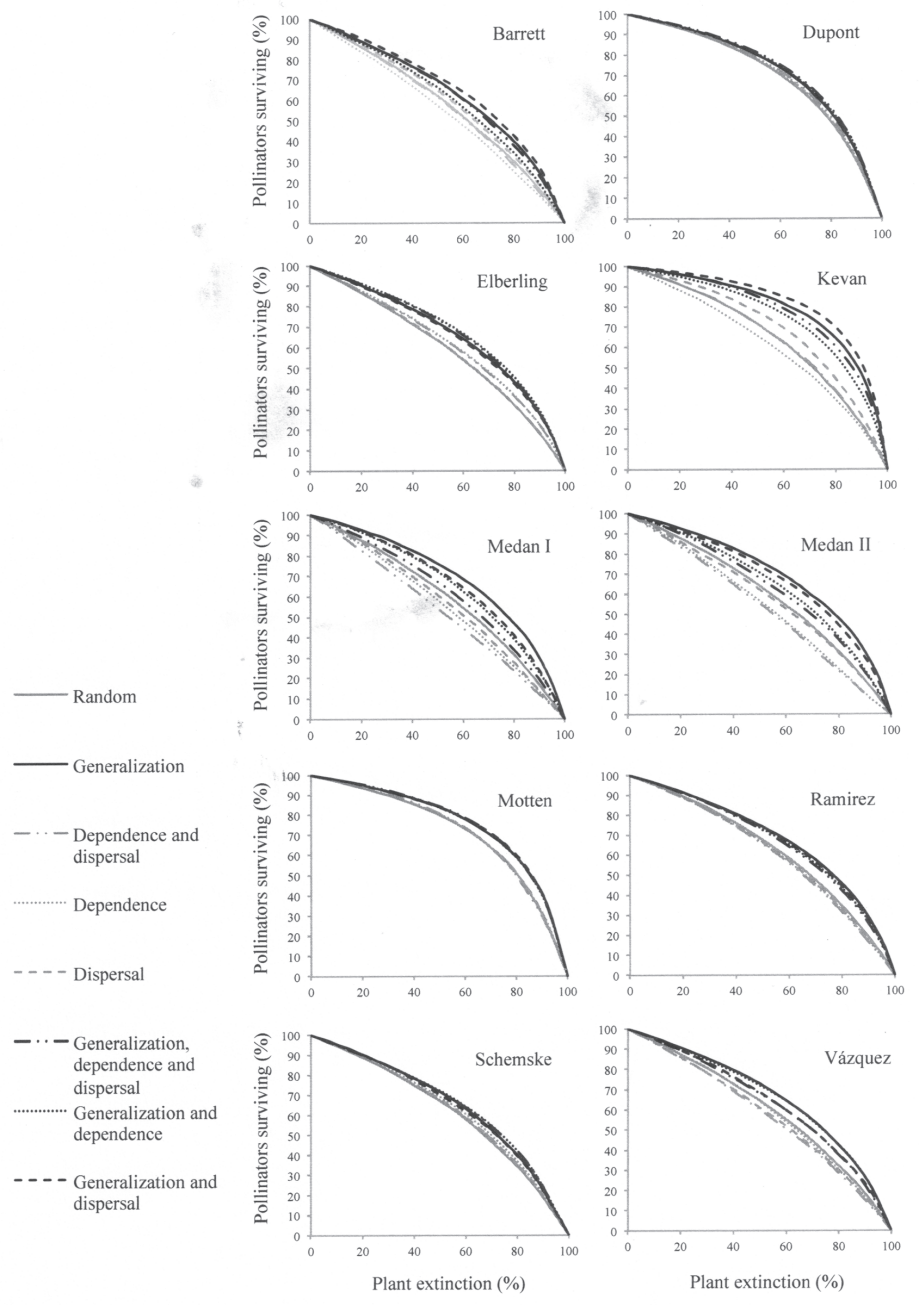
Plant sensitivity to pollinator loss and network robustness. Proportion of pollinator species surviving under different scenarios of plant extinction representing plant sensitivity to pollinator loss in 10 plant-pollinator networks. Scenarios where plant extinction probability was linked to plant generalization (with or without taking into account other traits) were drawn in black. Scenarios that did not consider plant generalization into plant extinction probability were drawn in grey.

The proportion of pollinator species surviving after plant extinction followed a slow-decaying curve under most of the scenarios (Fig. 2). The robustness of plant-pollinator networks varied depending on which plant trait or combination of traits influenced plant probability of extinction (Fig. 2; S5–S12 Tables). In most assemblages (80%), networks were more robust when the sequence of extinctions depended on differences in generalization among plant species whether or not the other biological traits affected extinction probabilities (Fig. 2; S5–S12 Tables). Seven networks (70%) showed lower robustness when all species had the same probability of extinction (random scenario) and under scenarios where plant loss was influenced only by plant dependence on pollinators, dispersal ability or these two traits (80%; Fig. 2). In two networks (Schemske and Dupont) robustness was similar among all scenarios and in two networks (Ramirez and Vázquez) robustness under the random scenario was similar to robustness observed under the scenario considering plant generalization and the two biological traits (Fig. 2; S6 and S12–S14 Tables).

Differences in the robustness of networks among the set of scenarios where plant generalization influenced the sequence of extinctions varied across assemblages. Most networks (80%) showed similar robustness to extinction sequences determined by the combination of the three traits and to extinctions only based on plant generalization (Fig. 2; S5–S8, S11–S14 Tables). Only two networks were less robust when the three traits were considered (Medan I and Medan II; Fig. 2; S9 and S10 Tables).

Differences in the robustness of networks between the random scenario and the set of scenarios where extinction sequences were determined by plant dependence and/or dispersal also varied across assemblages. Half of assemblages showed similar robustness between scenarios where extinction probabilities were associated to either one or both biological traits and random scenarios (Barrett, Dupont, Elberling, Motten and Vázquez; Fig. 2; S5–S7, S11 and S14 Tables). The scenario considering plant dispersal and dependence on pollinators led to less robust networks than the random scenario in three assemblages (Medán I, Medán II and Ramirez; Fig. 2; S9, S10 and S12 Tables).

## Discussion

Focusing on how traits determining species sensitivity to partner loss may influence species patterns of interaction may improve our understanding of the impacts of species loss on assemblage maintenance [6, 7]. Contrary to what we hypothesized, generalist plants were not more sensitive to pollinator loss. We showed that low dispersal plants interacted with more generalized pollinators and that the contribution of plants to nestedness was associated with the interaction among biological traits. Slightly dependent low dispersal plants were those contributing more to nestedness whereas species showing the highest sensitivity to pollinator loss were among those species showing the lowest contribution. Finally, in most assemblages (80%) considering plant generalization and biological traits as determinants of species probability of extinction led to networks of similar robustness to scenarios only including plant generalization. Below, we discuss the implications of our results in light of how plant species may persist locally and how this persistence will maintain plant-pollinator assemblages.

### Plant interaction patterns and sensitivity to pollinator loss

Since functional redundancy among pollinators may lead to less fluctuating pollination service in generalist plants [15–17, 49], species depending more on pollinators to produce seeds (e.g. dioecious or self-incompatible species) are expected to be more generalists. Alternatively, to cope with fluctuations in pollinator service species depending more on pollinators may be expected to be better dispersers [32], as a way to buffer local pollination limitation. Although a good ability to colonize has traditionally been associated with low dependence on pollinators—i.e., self-compatibility or autonomous self-pollination [30, 31]—, the association between strong dependence on pollinators and high dispersal has been predicted by theoretical studies and both trends have been observed in empirical studies [32, 34, 50]. Our results showed that plants more sensitive to pollinator loss did not showed higher ecological generalization on pollinators. Species with strong dependence on pollinators and high dispersal ability might persist under unfavorable pollination environments by receiving seeds from other sites whereas low-dispersal plants with autonomous self-pollination mechanisms might assure their reproduction by performing self-pollination.

Moreover, low-dispersal plants interacted with more generalized pollinators which might fluctuate less in abundance and thus may provide a more reliable pollination service [15, 26, 51]. Thus, the interaction with pollinators that visit multiple plant species—a pervasive pattern in pollination networks [20]—might also be an alternative pathway for the persistence of low-dispersal plants. However, the interaction with more generalized pollinators might also increase the arrival of heterospecific pollen to stigmas, negatively affecting the reproduction of plants [52, 53]. The quantity of heterospecific pollen delivered by generalist pollinators might depend on how pollinator foraging behavior can be affected by the more likely patchy distribution of low dispersal plants [54].

Higher overlap of interactions with other species may also increase species persistence [55]. An indirect positive effect may exist among plants interacting with the same pollinators since the persistence of any of the plant species may allow the persistence of pollinators and thus maintain the pollination service of other plants [56]. The highest contribution to nestedness was observed in plants with slight dependence on pollinators, i.e. plants that can reproduce even when pollinators are scarce. Therefore, the persistence of slightly dependent plants despite temporal fluctuations of pollinator abundance might facilitate the persistence of more sensitive plants. However, we also found that plants having the highest sensitivity to pollinator loss—i.e., strongly dependent, low-dispersal species—where among the groups of plants showing the lowest contribution to nestedness. Ecological specialization between interacting partners may contribute to increase the vulnerability of interactions to disruption [57], which might compromise the persistence of these highly sensitive plants and their pollinators.

### Plant sensitivity to pollinator loss and network robustness

Plant-pollinator assemblages may show high robustness on average to the random extinction of species, but face higher fragility when generalists are lost earlier in the sequence of extinctions [11, 13]. We assessed network robustness under scenarios of plant extinction representing plant sensitivity to pollinator loss associated with two key biological traits: plant dependence on pollinators and dispersal ability. In accordance with previous studies [11, 13], the early collapse of networks was not observed under the different scenarios of species extinction. Thus, plant-pollinator networks may be tolerant to the loss of more sensitive plants. However, this tolerance relies on the assumption that pollinators are functionally redundant which is still unknown for most of plant-pollinator networks [13].

Since generalization implies redundancy of interaction partners, more robust assemblages can be expected when extreme generalists are the least likely to be lost [11, 13]. The higher robustness of scenarios where extinction sequences depended on plant generalization compared to scenarios where generalization was not considered is in agreement with that expectation. Moreover, the lower robustness of networks under scenarios where extinction order depended only on either plant dependence on pollinators or dispersal ability may be explained by the earlier removal of generalized plants. The early extinction of generalists lies with the similar generalization levels we found among plants with different dependence on pollinators and dispersal ability, i.e. generalist plants may show both high and low sensitivity to pollinator loss. However, as the nested structure of interactions may explain the robustness of plant-pollinator assemblages [11, 13, 56, 58, 59], differences in network robustness among scenarios might be associated, indeed, to differences in contribution to nestedness among species. When extinction sequences depended on dispersal, plants being removed earlier may decrease network nestedness, as low-dispersal (more sensitive) plants were those contributing more to the nested structure. The interaction of strongly dependent, low-dispersal plants with more particular sets of pollinators may explain why scenarios considering dependence on pollinators with or without dispersal ability led to less robust networks than scenarios including plant generalization. Contribution to nestedness may partially increase with species ecological generalization [9], which may explain why scenarios where generalists were less likely to be lost were more robust.

Plant sensitivity to pollinator loss has been included into simulated scenarios of plant extinction in two previous studies, by using plant-pollinator frequency of interactions as a surrogate of species dependence on interaction partners [13, 60]. By removing either pollinators or plants, Kaiser-Bunbury et al. [13] showed that the early removal of most important interaction partners (i.e. with the strongest interaction frequencies) led to less robust networks than scenarios assuming the early removal of generalists. Vieira & Almeida-Neto [60] included plant dependence on pollinators into extinction scenarios by considering interaction frequency among plants and pollinators, and by assigning different levels of dependence on pollinators to whole plant communities of real networks (i.e. plants of the same community had the same ability to self-pollinate). They showed that lower mean plant ability to self-pollinate increased the number of co-extinctions per extinction event, decreasing network robustness to the loss of generalists [60]. In contrast, we found that when breeding system, dispersal ability and plant generalization influenced the sequences of plant extinction only two networks showed lower robustness than under the generalization scenario. Thus, one of the next questions to be addressed is to what extent adding pollinator effectiveness may evidence the influence of biological traits on network robustness. Asymmetric dependencies seem to be the rule in mutualistic networks [61, 62] and species importance for interaction partners (i.e. species strength) has been reported to be positively associated with species generalization [61]. Thus, we hypothesize that the extinction of more sensitive plants does not cause the co-extinction of shared pollinators and other plants, because highly sensitive plants interacted with more particular sets of pollinators. However, we recognize that the influence of pollinator effectiveness on plant persistence and thus on community robustness remains to be empirically tested.

### Conclusions and Future Directions

Our results suggest that networks might be tolerant to pollinator loss because plants that are central to network organization may have alternative strategies to cope with pollen limitation, which may allow their persistence under unfavorable pollinator environments. Thus, focusing on network organization as a determinant of assemblage robustness might be a good approximation to estimate the fragility of plant-pollinator networks to species loss. However, more complete understanding of the importance of plant sensitivity to pollinator loss should be achieved by studying the effects of functional redundancy within pollinators of more generalist species, including how per-visit effectiveness and species local abundance relate to each other and with temporal fluctuation in pollinator abundance, three important features that may influence species generalization level [15]. Moreover, although the nested structure of plant-pollinator assemblages may provide higher metacommunity robustness to habitat loss [63], more sensitive plants interacting with more particular pollinator assemblages may be more prone to be lost. Since plant breeding system and dispersal ability may modulate plant response to habitat fragmentation [24, 64], future studies assessing how changes in landscape configuration affect the relationships between plant interaction patterns and plant sensitivity to pollinator loss may improve our understanding of the effects of one of the major threats to biodiversity.

We thank Silvana Buzato, Pedro Jordano, Miguel Fortuna and the Guimarães (Miúdo) Lab for discussions on the ideas presented in this manuscript. To Alfredo Valido, Alicia Sérsic, Andrea Cosacov and Nelson Ramirez for providing information and specific bibliography on species traits. To Alejandra Tapia for advice with statistical analyses. To Lauren Ponisio and three anonymous reviewers for comments on the manuscript.

## Supporting Information

S1 Table General information of the plant-pollinator networks from the Interaction Web Database used in this study. Network, Habitat type, localization, total number of plant and pollinator species (Sp), connectance (c), degree of nestedness (NODF), and percentage of plant species with information on dependence on pollinators and dispersal ability (% Plants DP & DA) are shown for each network. References as stated at the IWDB are also shown. Networks with their original name in italic are those that were used in simulations of plant extinction. For abbreviation purposes we used the surname of the first author to refer to each network.
(PDF)

S2 Table Plant interaction patterns and sensitivity to pollinator loss. Network, species, family, dependence on pollinators (DP), dispersal ability (DA), plant generalization, contribution to nestedness (nestedness) and mean generalization of pollinators of the plant species used in this study. Data on plant generalization, nestedness and mean generalization of pollinators were obtained as described in the M&M section. The first citation appearing in the column “Reference” refers to the bibliographic source from which data on dependence on pollinators was extracted and the second one refers to data on dispersal ability. Only one reference is presented when information on both traits was extracted from the same source. Three references are shown when more than one reference was available for one of the traits. “Strong”, “Inter” and “Slight” refer to strongly, intermediately and slightly dependent plants, respectively, in the column referring to dependence on pollinators (DP). “Low” and “High” refer to high and low-dispersal plants, respectively, in the column referring to dispersal ability (DA).
(PDF)

S3 Table Plant sensitivity to pollinator loss and network robustness. Scenarios linking plant probability of extinction to different plant traits associated with plant sensitivity to pollinator loss (i.e. plant generalization, dependence on pollinators, dispersal ability) or combinations of these traits. The random scenario (no trait is considered) was also explored.
(PDF)

S4 Table Plant interaction patterns and sensitivity to pollinator loss: partition of variation and permutation analyses results. Sum of squares (SS), degrees of freedom (df), mean squares (MS), and F-ratio are shown for plant generalization on pollinators, contribution to nestedness and mean pollinator generalization. Probability values (p) obtained from permutation analyses are also shown.
(PDF)

S5 Table Plant sensitivity to pollinator loss and network robustness. Mean differences of robustness (Mean) between pairs of scenarios, and minimum (CI min) and maximum (CI max) limits of the 95% confidence interval of each difference are shown for the Barrett network. Pairwise comparisons were planned a-priori. Scenarios: dependence on pollinators (DP), dispersal ability (DA), dependence on pollinators and dispersal ability (DPDA), random (R), dependence on pollinators and generalization (DPG), dispersal ability and generalization (DAG), dependence on pollinators, dispersal ability and generalization (DPDAG) and generalization (G).
(PDF)

S6 Table Plant sensitivity to pollinator loss and network robustness. Mean differences of robustness (Mean) between pairs of scenarios, and minimum (CI min) and maximum (CI max) limits of the 95% confidence interval of each difference are shown for the Dupont network. Pairwise comparisons were planned a-priori. Scenarios: dependence on pollinators (DP), dispersal ability (DA), dependence on pollinators and dispersal ability (DPDA), random (R), dependence on pollinators and generalization (DPG), dispersal ability and generalization (DAG), dependence on pollinators, dispersal ability and generalization (DPDAG) and generalization (G).
(PDF)

S7 Table Plant sensitivity to pollinator loss and network robustness. Mean differences of robustness (Mean) between pairs of scenarios, and minimum (CI min) and maximum (CI max) limits of the 95% confidence interval of each difference are shown for the Elberling network. Pairwise comparisons were planned a-priori. Scenarios: dependence on pollinators (DP), dispersal ability (DA), dependence on pollinators and dispersal ability (DPDA), random (R), dependence on pollinators and generalization (DPG), dispersal ability and generalization (DAG), dependence on pollinators, dispersal ability and generalization (DPDAG) and generalization (G).
(PDF)

S8 Table Plant sensitivity to pollinator loss and network robustness. Mean differences of robustness (Mean) between pairs of scenarios, and minimum (CI min) and maximum (CI max) limits of the 95% confidence interval of each difference are shown for the Kevan network. Pairwise comparisons were planned a-priori. Scenarios: dependence on pollinators (DP), dispersal ability (DA), dependence on pollinators and dispersal ability (DPDA), random (R), dependence on pollinators and generalization (DPG), dispersal ability and generalization (DAG), dependence on pollinators, dispersal ability and generalization (DPDAG) and generalization (G).
(PDF)

S9 Table Plant sensitivity to pollinator loss and network robustness. Mean differences of robustness (Mean) between pairs of scenarios, and minimum (CI min) and maximum (CI max) limits of the 95% confidence interval of each difference are shown for the Medán II network. Pairwise comparisons were planned a-priori. Scenarios: dependence on pollinators (DP), dispersal ability (DA), dependence on pollinators and dispersal ability (DPDA), random (R), dependence on pollinators and generalization (DPG), dispersal ability and generalization (DAG), dependence on pollinators, dispersal ability and generalization (DPDAG) and generalization (G).
(PDF)

S10 Table Plant sensitivity to pollinator loss and network robustness. Mean differences of robustness (Mean) between pairs of scenarios, and minimum (CI min) and maximum (CI max) limits of the 95% confidence interval of each difference are shown for the Medán I network. Pairwise comparisons were planned a-priori. Scenarios: dependence on pollinators (DP), dispersal ability (DA), dependence on pollinators and dispersal ability (DPDA), random (R), dependence on pollinators and generalization (DPG), dispersal ability and generalization (DAG), dependence on pollinators, dispersal ability and generalization (DPDAG) and generalization (G).
(PDF)

S11 Table Plant sensitivity to pollinator loss and network robustness. Mean differences of robustness (Mean) between pairs of scenarios, and minimum (CI min) and maximum (CI max) limits of the 95% confidence interval of each difference are shown for the Motten network. Pairwise comparisons were planned a-priori. Scenarios: dependence on pollinators (DP), dispersal ability (DA), dependence on pollinators and dispersal ability (DPDA), random (R), dependence on pollinators and generalization (DPG), dispersal ability and generalization (DAG), dependence on pollinators, dispersal ability and generalization (DPDAG) and generalization (G).
(PDF)

S12 Table Plant sensitivity to pollinator loss and network robustness. Mean differences of robustness (Mean) between pairs of scenarios, and minimum (CI min) and maximum (CI max) limits of the 95% confidence interval of each difference are shown for the Ramirez network. Pairwise comparisons were planned a-priori. Scenarios: dependence on pollinators (DP), dispersal ability (DA), dependence on pollinators and dispersal ability (DPDA), random (R), dependence on pollinators and generalization (DPG), dispersal ability and generalization (DAG), dependence on pollinators, dispersal ability and generalization (DPDAG) and generalization (G).
(PDF)

S13 Table Plant sensitivity to pollinator loss and network robustness. Mean differences of robustness (Mean) between pairs of scenarios, and minimum (CI min) and maximum (CI max) limits of the 95% confidence interval of each difference are shown for the Schemske network. Pairwise comparisons were planned a-priori. Scenarios: dependence on pollinators (DP), dispersal ability (DA), dependence on pollinators and dispersal ability (DPDA), random (R), dependence on pollinators and generalization (DPG), dispersal ability and generalization (DAG), dependence on pollinators, dispersal ability and generalization (DPDAG) and generalization (G).
(PDF)

S14 Table Plant sensitivity to pollinator loss and network robustness. Mean differences of robustness (Mean) between pairs of scenarios, and minimum (CI min) and maximum (CI max) limits of the 95% confidence interval of each difference are shown for the Vazquez network. Pairwise comparisons were planned a-priori. Scenarios: dependence on pollinators (DP), dispersal ability (DA), dependence on pollinators and dispersal ability (DPDA), random (R), dependence on pollinators and generalization (DPG), dispersal ability and generalization (DAG), dependence on pollinators, dispersal ability and generalization (DPDAG) and generalization (G).
(PDF)

S1 Fig Plant contribution to nestedness and dispersal ability. Box-plots of plant contribution to nestedness of species with different dispersal ability. Black lines within boxes represent median values. Upper and lower limits of boxes represent 1^st^ and 3^rd^ quartiles, respectively. Boxes were drawn with widths proportional to the number of observations in each group. “Low” and “High” refer to low and high-dispersal plants, respectively.
(TIFF)
