# Supplementary material for: The Robustness of Plant-Pollinator Assemblages: Linking Plant Interaction Patterns and Sensitivity to Pollinator Loss
Source: PLoS One. 2015 Feb 3;10(2):e0117243. doi: 10.1371/journal.pone.0117243 (PMC4315602; doi:10.1371/journal.pone.0117243)
Supplement: S1 Table — Network, Habitat type, localization, total number of plant and pollinator species (Sp), connectance (c), degree of nestedness (NODF), and percentage of plant species with information on dependence on pollinators and dispersal ability (% Plants DP & DA) are shown for each network. References as stated at the IWDB are also shown. Networks with their original name in italic are those that were used in simulations of plant extinction. For abbreviation purposes we used the surname of the first author to refer to each network. (PDF) [file pone.0117243.s001.pdf]

Network	Habitat type	Localization	Sp	Sa	c	NODF	% Plants DP&DA	Reference
Schemske	Maple-oak woodland	USA	7	32	0.26	53.4	100%	Schemske DW, Willson MF, Melampy MN, Miller LJ, Verner L, et al. (1978) Flowering ecology of some spring woodland herbs. Ecology 59: 351-366.
Vazquez	Evergreen montane forest	Argentina	10	90	0.18	30.01	100%	Vázquez DP (2002) Interactions among Introduced Ungulates, Plants, and Pollinators: A Field Study in the Temperate Forest of the Southern Andes. Doctoral Dissertation thesis, University of Tennessee, Knoxville, Tennessee, USA. Vázquez DP, Simberloff D (2002) Ecological specialization and susceptibility to disturbance:

								conjectures and refutations. *Am Nat* 159: 606-623. Vázquez DP, Simberloff D (2003) Changes in interaction biodiversity induced by an introduced ungulate. Ecol Lett 6: 1077-1083.
Dupont	High-altitude desert	Tenerife	11	38	0.25	35.58	63%	Dupont YL, Hansen DM, Olesen JM (2003) Structure of a plant-flower-visitor network in the high-altitude sub-alpine desert of Tenerife, Canary Islands. Ecography 26: 301-310.
Barrett	Boreal Forest	Canada	12	112	0.11	30.78	100%	Barrett SCH, Helenurm K (1987) The reproductive-

								biology of boreal forest herbs.1. Breeding systems and pollination. *Can J Bot* 65: 2036-2046.
Motten	Deciduous forest	USA	13	44	0.25	51.26	92%	Motten AF (1982) Pollination ecology of the spring wildflower community in the deciduous forests of piedmont North Carolina. Doctoral Dissertation thesis, Duke University, Duhram, North Carolina, USA.<br>Motten AF (1986) Pollination ecology of the spring wildflower community of a temperate deciduous forest. Ecol Monogr 56: 21-42.
Medan I	Xeric scrub	Laguna Diamante, Mendoza, Argentina	21	45	0.09	18.02	100%	Medan D, Montaldo NH, Devoto M, Mantese A, Vasellati V, et al. (2002) Plant-pollinator relationships at two altitudes in the Andes of Mendoza, Argentina. Arctic Antarctic Alpine Res

								34: 233-241.
Medan II	Xeric scrub	Rio Blanco, Mendoza, Argentina	23	72	0.08	22.88	61%	Medan D, Montaldo NH, Devoto M, Mantese A, Vasellati V, et al. (2002) Plant-pollinator relationships at two altitudes in the Andes of Mendoza, Argentina. Arctic Antarctic Alpine Res 34: 233-241.
Elbering	Alpine subarctic community	Sweden	23	118	0.09	15.28	91%	Elberling H, Olesen JM (1999) The structure of a high latitude plant-flower visitor system: the dominance of flies. Ecography 22: 314-323.
Ramirez	Palm swam community	Venezuela	28	53	0.07	11.16	79%	Ramirez N, Brito Y (1992) Pollination biology in a palm swamp community in the venezuelan central plains. Bot J Linn Soc 110: 277-302.
Kevan	High Artic	Canada	30	115	0.09	36.36	70%	Kevan PG (1970) High arctic insect-flower visitor relations: the inter-relationships of arthropods and

								flowers at Lake Hazen, Ellesmere Island, Northwest Territories, Canada. Ph.D. thesis thesis, University of Alberta.
Inouye	Montane forest	Australia	41	91	0.08	17.21	44%	Inouye DW, Pyke GH (1988) Pollination biology in the Snowy Mountains of Australia: comparisons with montane Colorado, USA. Austr J Ecol 13: 191-210.
Kalin Arroyo III	Andean scrub	Chile	41	28	0.08	20.48	22%	Arroyo MTK, Primack RB, Armesto JJ (1982) Community studies in pollination ecology in the high temperate Andes of Central Chile. I. Pollination mechanisms and altitudinal variation. Am J Bot 69: 82-97.
Kalin Arroyo I	Andean scrub	Chile	87	98	0.04	15.11	28%	Arroyo MTK, Primack RB, Armesto JJ (1982) Community studies in pollination ecology in the

high temperate Andes of Central Chile. I.

Pollination mechanisms and altitudinal variation.

Am J Bot 69: 82-97.
