# Supplementary material for: The Robustness of Plant-Pollinator Assemblages: Linking Plant Interaction Patterns and Sensitivity to Pollinator Loss
Source: PLoS One. 2015 Feb 3;10(2):e0117243. doi: 10.1371/journal.pone.0117243 (PMC4315602; doi:10.1371/journal.pone.0117243)
Supplement: S2 Table — Network, species, family, dependence on pollinators (DP), dispersal ability (DA), plant generalization, contribution to nestedness (nestedness) and mean generalization of pollinators of the plant species used in this study. Data on plant generalization, nestedness and mean generalization of pollinators were obtained as described in the M&M section. The first citation appearing in the column “Reference” refers to the bibliographic source from which data on dependence on pollinators was extracted and the second one refers to data on dispersal ability. Only one reference is presented when information on both traits was extracted from the same source. Three references are shown when more than one reference was available for one of the traits. “Strong”, “Inter” and “Slight” refer to strongly, intermediately and slightly dependent plants, respectively, in the column referring to dependence on pollinators (DP). “Low” and “High” refer to high and low-dispersal plants, respectively, in the column referring to dispersal ability (DA). (PDF) [file pone.0117243.s002.pdf]

Network	Species	Family	DP	DA	Plant Generalization	Nestedness	Mean generalization pollinators	Reference
Barrett	Cornus canadensis L.	Cornaceae	High	High	0,64	0,22	0,14	Barrett S, Helenurm K (1987) The reproductive biology of boreal forest herbs. Breeding systems and pollination. Can J Bot 65: 2036–2046.
Barrett	Maianthemum canadense Desf.	Asparagaceae	High	High	0,25	0,22	0,23	Barrett S, Helenurm K (1987) The reproductive biology of boreal forest herbs. Breeding systems and pollination. Can J Bot 65: 2036–2046.
Barrett	Linnaea borealis L.	Caprifoliaceae	Low	Low	0,21	0,21	0,24	Barrett S, Helenurm K (1987) The reproductive biology of boreal forest herbs. Breeding systems and pollination. Can J Bot 65: 2036–2046.<br>Thompson, K, Band S, Hodgson JG (1993) Seed size and shape predict persistence. Funct Ecol 7: 236–241.<br>Frerker K, Sonnier G, Waller DM (2013) Browsing rates and ratios provide reliable indices of ungulate impacts on forest plant communities. For Ecol Manage 291: 55–64.
Barrett	Aralia nudicaulis L.	Araliaceae	High	High	0,17	0,19	0,25	Barrett S, Helenurm K (1987) The reproductive biology of boreal forest herbs. Breeding systems and pollination. Can J Bot 65: 2036–2046.<br>Frerker K, Sonnier G, Waller DM (2013) Browsing rates and ratios provide reliable indices of ungulate impacts on forest plant communities. For Ecol Manage 291: 55–64.
Barrett	Clintonia borealis (Aiton) Raf.	Liliaceae	Low	High	0,15	0,18	0,26	Barrett S, Helenurm K (1987) The reproductive biology of boreal forest herbs. Breeding systems and pollination. Can J Bot 65: 2036–2046.
Barrett	Oxalis acetosella L.	Oxalidaceae	Low	Low	0,06	0,12	0,28	Barrett S, Helenurm K (1987) The reproductive biology of boreal forest herbs. Breeding systems and pollination. Can J Bot 65: 2036–2046.<br>Jasieniuk M, Lechowicz M (1987) Spatial and Temporal Variation in Chasmogamy and Cleistogamy in Oxalis montana (Oxalidaceae). Am J Bot 74: 1672–1680.
Barrett	Orthilia secunda (L.) House	Ericaceae	Low	High	0,04	0,15	0,38	Barrett S, Helenurm K (1987) The reproductive biology of boreal forest herbs. Breeding systems and pollination. Can J Bot 65: 2036–2046.<br>http://www.seed-dispersal.info
Barrett	Trillium undulatum Willd.	Melanthiaceae	Low	Low	0,04	0,16	0,42	Barrett S, Helenurm K (1987) The reproductive biology of boreal forest herbs. Breeding systems and pollination. Can J Bot 65: 2036–2046.<br>http://data.kew.org/sid/
Barrett	Chimaphila umbellata (L.) Nutt.	Ericaceae	Low	Low	0,03	0,16	0,39	Barrett S, Helenurm K (1987) The reproductive biology of boreal forest herbs. Breeding systems and pollination. Can J Bot 65: 2036–2046.<br>http://data.kew.org/sid/
Barrett	Trientalis borealis Raf.	Primulaceae	High	Low	0,03	0,11	0,31	Barrett S, Helenurm K (1987) The reproductive biology of boreal forest herbs. Breeding systems and pollination. Can J Bot 65: 2036–2046.<br>Anderson RC, Beare M H (1983) Breeding System and Pollination Ecology of Trientalis borealis (Primulaceae). Am J Bot 70: 408–415.
Barrett	Cypripedium acaule Aiton	Orchidaceae	Inter	High	0,02	0,23	0,54	Barrett S, Helenurm K (1987) The reproductive biology of boreal forest herbs. Breeding systems and pollination. Can J Bot 65: 2036–2046.<br>Arditti J, Michaud J, Healey P (1979) Morphometry of Orchid seeds. I. Paphiopedilum and native California and related species of cypripedium. 66: 1128–1137.
Barrett	Medeola virginiana L.	Liliaceae	High	High	0,01	0,00	0,08	Barrett S, Helenurm K (1987) The reproductive biology of boreal forest herbs. Breeding systems and pollination. Can J Bot 65: 2036–2046.
Dupont	Echium wildpretii H.Pearson ex Hook.f.	Boraginaceae	Low	Low	0,47	0,22	0,32	Dupont YL, Hansen DM, Olesen JM (2003) Structure of a plant – flower-visitor network in the high-altitude sub-alpine desert of Tenerife , Canary Islands. 3: 301–310.<br>Valido A, Rodríguez-Rodríguez C, Jordano P (2011) Interacciones entre plantas y polinizadores en el parque nacional de Teide: consecuencias ecológicas de la introducción masiva de la abeja doméstica (*Apis mellifera*, Apidae). In: Ramírez L, Asensio B, editors. Proyectos de investigación en parques nacionales: 2007-2010. pp. 205-232.
Dupont	Pimpinella cumbrae Link	Apiaceae	Low	High	0,37	0,17	0,32	Calero A, Santos A (1986) Reproductive biology of the high altitude canarian flora. 5th OPTIMA meeting, Istambul.<br>Real J, Carqué E, Bañares A, Marrero MV (2001) Morphological study of fruits, seeds and seedlings of some supracanarian dry bioclimatic belt endemic plants. Vieraea 29: 1-15.
Dupont	Erysimum scoparium (Brouss. ex Willd.) Wettst.	Brassicaceae	Low	Low	0,26	0,22	0,40	Valido A, Rodríguez-Rodríguez C, Jordano P (2011) Interacciones entre plantas y polinizadores en el parque nacional de Teide: consecuencias ecológicas de la introducción masiva de la abeja doméstica (Apis mellifera, Apidae). In: Ramírez L, Asensio B, editors. Proyectos de investigación en parques nacionales: 2007-2010. pp. 205-232.
Dupont	Spartocytisus supranubius (L. f.) Christ ex G. Kunkel	Leguminosae	High	High	0,24	0,19	0,41	Calero A, Santos A (1986) Reproductive biology of the high altitude canarian flora. 5th OPTIMA meeting, Istambul.<br>Valido A, Rodríguez-Rodríguez C, Jordano P (2011) Interacciones entre plantas y polinizadores en el parque nacional de Teide: consecuencias ecológicas de la introducción masiva de la abeja doméstica (Apis mellifera, Apidae). In: Ramírez L, Asensio B, editors. Proyectos de investigación en parques nacionales: 2007-2010. pp. 205-232.
Dupont	Tolpis webbii Sch.Bip.	Compositae	High	High	0,21	0,19	0,42	Crawford DJ, Archibald JK, Kelly JK, Mort ME, Santos-Guerra A (2010) Mixed mating in the "obligately outcrossing" Tolpis (Asteraceae) of the Canary Islands. Plant Species Biol 25: 114–119.<br>Pérez de Paz J, Caujapé-Castells J (2013) A review of the allozyme data set for the Canarian endemic flora: causes of the high genetic diversity levels and implications for conservation. Ann Bot 111: 1059–1073.
Dupont	Scrophularia glabrata Aiton	Scrophulariaceae	Low	Low	0,18	0,13	0,34	Valido A, Rodríguez-Rodríguez C, Jordano P (2011) Interacciones entre plantas y polinizadores en el parque nacional de Teide: consecuencias ecológicas de la introducción masiva de la abeja doméstica (Apis mellifera, Apidae). In: Ramírez L, Asensio B, editors. Proyectos de investigación en parques nacionales: 2007-2010. pp. 205-232.
Dupont	Adenocarpus viscosus (Willd.) Webb & Berthel.	Leguminosae	High	Low	0,05	0,16	0,41	Calero A, Santos A (1986) Reproductive biology of the high altitude canarian flora. 5th OPTIMA meeting, Istambul.<br>Arévalo JR, Fernández-Palacios JM (2005) Gradient analysis of exotic Pinus radiata plantations and potential restoration of natural vegetation in Tenerife, Canary Islands (Spain). Acta Oecologica 27: 1–8.
Eberling	Dryas octopetala L.	Rosaceae	High	High	0,24	0,15	0,15	Mcgraw AJB, Antonovics J (1983) Experimental Ecology of Dryas Octopetala Ecotypes : I . Ecotypic Differentiation and Life-Cycle Stages of Selection. 71: 879–897.
Eberling	Diapensia lapponica L.	Diapensiaceae	Low	High	0,21	0,08	0,12	Molau U (1993) Relationships between flowering phenology and Life History in Relationships Flowering Phenology Strategies in Tundra Plants. Arct Alp Res 25: 391–402.<br>Densmore R (1997) Effect of day length on germination of seeds collected in Alaska. Am J Bot 84: 274.
Eberling	Saxifraga aizoides L.	Saxifragaceae	Inter	Low	0,21	0,08	0,11	Molau U (1993) Relationships between flowering phenology and Life History in Relationships Flowering Phenology Strategies in Tundra Plants. Arct Alp Res 25: 391–402.<br>Meier C, Holderegger R (1998) Breeding system, germination, and phenotypic differences among populations of Saxifraga aizoides (Saxifragaceae) at the periphery of its alpine distribution. Nord J Bot 18: 681–688.<br>Ninot JM, Grau O, Carrillo E, Guàrdia R, Lluent A, et al. (2012) Functional Plant Traits and Species Assemblage in Pyrenean Snowbeds. Folia Geobot 48: 23–38.
Eberling	Potentilla crantzii (Crantz) Beck ex Fritsch	Rosaceae	Low	Low	0,19	0,20	0,19	Dobeš C, Milosevic a, Prohaska D, Scheffknecht S, Sharbel TF, et al. (2013) Reproductive differentiation into sexual and apomictic polyploid cytotypes in Potentilla puberula (Potentilleae, Rosaceae). Ann Bot 112: 1159–1168.<br>Welling P, Laine K (2002) Regeneration by seeds in alpine meadow and heath vegetation in sub-arctic Finland. J Veg Sci 13: 217–226.
Eberling	Silene acaulis (L.) Jacq.	Caryophyllaceae	Inter	Low	0,13	0,12	0,17	Morris W, Doak D (1998) Life history of the long-lived gynodioecious cushion plant Silene acaulis (Caryophyllaceae), inferred from size-based population projection matrices. Am J Bot 85: 784.<br>Delph LF, Carroll SB (2001) Factors affecting relative seed fitness and female frequency in a gynodioecious species, Silene acaulis. Evol Ecol Res 3: 487–505.

Eberling	Parnassia palustris L.	Celastraceae	Low	High	0,11	0,05	0,13	Bossuyt B (2007) Genetic rescue in an isolated metapopulation of a naturally fragmented plant species, Parnassia palustris. Conserv Biol 21: 832–841.
Eberling	Persicaria vivipara (L.) Ronse Decr.	Polygonaceae	Inter	High	0,11	0,13	0,23	Honnay O, Jacquemyn H (2007) A meta-analysis of the relation between mating system, growth form and genotypic diversity in clonal plant species. Evol Ecol 22: 299–312.<br>Welling P, Laine K (2002) Regeneration by seeds in alpine meadow and heath vegetation in sub-arctic Finland. J Veg Sci 13: 217–226.
Eberling	Saxifraga oppositifolia L.	Saxifragaceae	Inter	High	0,08	0,08	0,17	Gugerli F (1997) Sexual Reproduction in Saxifraga oppositifolia L . and Saxifraga biflora All . (Saxifragaceae) in the Alps. Int J Plant Sci 158: 274–281.<br>Holderegger R, Stehlik I, Abbott RJ (2002) Molecular analysis of the Pleistocene history of Saxifraga oppositifolia in the Alps. Mol Ecol 11: 1409–1418.
Eberling	Sedum roseum (L.) Scop.	Crassulaceae	Inter	High	0,06	0,04	0,14	Molau, U. 1993. Relationships between flowering phenology and life history strategies in tundra plants. *Arctic Alpine Res.* **25**, 391-402.<br>http://data.kew.org/sid/
Eberling	Salix lanata L.	Salicaceae	High	High	0,06	0,05	0,13	Totland ØR, Ottocornola MAS (2001) Pollen limitation of reproductive success in two sympatric alpine willows (Salicaceae) with contrasting pollination strategies. Am J Bot 88: 1011–1015.<br>Welling P, Laine K (2002) Regeneration by seeds in alpine meadow and heath vegetation in sub-arctic Finland. J Veg Sci 13: 217–226.
Eberling	Salix reticulata L.	Salicaceae	High	High	0,06	0,12	0,25	Culley TM, Weller SG, Sakai AK (2002) The evolution of wind pollination in angiosperms. 17: 361–369.<br>Welling P, Laine K (2002) Regeneration by seeds in alpine meadow and heath vegetation in sub-arctic Finland. J Veg Sci 13: 217–226.
Eberling	Trollius europaeus L.	Ranunculaceae	Inter	Low	0,06	0,06	0,17	Jaeger N, Després L (1998) Obligate mutualism between *Trollius europaeus* and its seed-parasite pollinators Chiastocheta flies in the Alps. Comptes Rendus de l'Académie des Sciences-Series III-Sciences de la Vie 321: 789-796.
Eberling	Pinguicula alpina L.	Lentibulariaceae	Inter	High	0,05	0,15	0,32	Molau U (1993) Relationships between flowering phenology and Life History in Relationships Flowering Phenology Strategies in Tundra Plants. Arct Alp Res 25: 391–402.<br>Degtjareva G, Casper J, Hellwig F, Sokoloff D (2004) Seed morphology in the genus *Pinguicula* (Lentibulariaceae) and its relation to taxonomy and phylogeny. Botanische Jahrbücher 125: 431-452.
Eberling	Rhododendron lapponicum (L.) Wahlenb.	Ericaceae	Inter	Low	0,04	0,09	0,22	Molau U (1993) Relationships between flowering phenology and Life History in Relationships Flowering Phenology Strategies in Tundra Plants. Arct Alp Res 25: 391–402.<br>http://www.flora.dempstercountry.org
Eberling	Salix polaris Wahlenb.	Salicaceae	High	High	0,04	0,05	0,15	Freeman D, Lovett Doust J, El-Keblawy A, Miglia K, McArthur E (1997) Sexual specialization and inbreeding avoidance in the evolution of dioecy. Bot Rev 63: 65–92.<br>Welling P, Laine K (2002) Regeneration by seeds in alpine meadow and heath vegetation in sub-arctic Finland. J Veg Sci 13: 217–226.
Eberling	Arabis alpina L.	Brassicaceae	High	High	0,03	0,10	0,25	Tedder a, Ansell SW, Lao X, Vogel JC, Mable BK (2011) Sporophytic self-incompatibility genes and mating system variation in Arabis alpina. Ann Bot 108: 699–713.<br>Gentili R, Armiraglio S, Rossi G, Sgorbati S, Baroni C (2010) Floristic patterns, ecological gradients and biodiversity in the composite channels (Central Alps, Italy). Flora - Morphol Distrib Funct Ecol Plants 205: 388–398.
Eberling	Astragalus alpinus L.	Leguminosae	High	Low	0,03	0,07	0,14	Kudo G, Molau ULF (1999) Variations in reproductive traits at inflorescence and flower levels of an arctic legume , Astragalus alpinus L .: Comparisons between a subalpine and an alpine population. Plant Species Biol 14: 181–191.<br>Welling P, Laine K (2002) Regeneration by seeds in alpine meadow and heath vegetation in sub-arctic Finland. J Veg Sci 13: 217–226.
Eberling	Cassiope tetragona (L.) D.Don	Ericaceae	Inter	Low	0,03	0,05	0,14	Molau U (1993) Relationships between flowering phenology and Life History in Relationships Flowering Phenology Strategies in Tundra Plants. Arct Alp Res 25: 391–402.<br>Welling P, Laine K (2002) Regeneration by seeds in alpine meadow and heath vegetation in sub-arctic Finland. J Veg Sci 13: 217–226.
Eberling	Harrimanella hypnoides (L.) Coville	Ericaceae	Inter	Low	0,02	0,12	0,28	Molau U (1993) Relationships between flowering phenology and Life History in Relationships Flowering Phenology Strategies in Tundra Plants. Arct Alp Res 25: 391–402.<br>Welling P, Laine K (2002) Regeneration by seeds in alpine meadow and heath vegetation in sub-arctic Finland. J Veg Sci 13: 217–226.
Eberling	Bartsia alpina L.	Orobanchaceae	Inter	High	0,01	0,02	0,09	Molau U, Eriksen B, Teilmann Knudsen J (1989) Predispersal seed predation in Bartsia alpina. Oecologia 81: 181–185.
Inouye	Senecio lautus G.Forst. ex Willd.	Compositae	High	High	0,14	0,21	0,21	Benson D, McDougall L (1994) Ecology of Sydney plant species part 2: dicotyledon families Asteraceae to Buddlejaceae. Cunninghamia 3: 789–1004.<br>Ali SI (1968) Senecio lautus complex in Australia IV. The biology of the complex. Phyton-Annales Rei Botanicae 13: 53–62.
Inouye	Hypochaeris radicata L.	Compositae	High	High	0,13	0,15	0,20	Picó FX, Ouborg NJ, Van Groenendael J (2004) Influence of selfing and maternal effects on life-cycle traits and dispersal ability in the herb *Hypochaeris radicata* (Asteraceae). Bot J Linn Soc 146: 163–170.
Inouye	Leucochrysum albicans var. tricolor (DC.) Paul G.Wilson	Compositae	High	High	0,13	0,11	0,13	Morgan JW, Meyer MJ, Young AG (2013) Severe habitat fragmentation leads to declines in genetic variation, mate availability, and reproductive success in small populations of a once-common australian grassland daisy. Int J Plant Sci 174: 1209-1218.
Inouye	Aciphylla glacialis (F.Muell.) Benth.	Apiaceae	High	High	0,12	0,12	0,15	Pickering CM (2001) Size and sex of floral displays affect insect visitation rates in the dioecious Australian alpine herb, *Aciphylla glacialis*. Nordic J Bot 21: 401–409.<br>Thorsen MJ, Dickinson KJM, Seddon PJ (2009) Seed dispersal systems in the New Zealand flora. Persp Plant Ecol Evol Syst 11: 285–309.
Inouye	Achillea millefolium L.	Compositae	High	High	0,08	0,06	0,10	Bourdot GW (1980) A study of the growth and development of yarrow (Achillea millefolium L.). Thesis. Lincoln College, University of Canterbury. Available from http://researcharchive.lincoln.ac.nz/handle/10182/1507
Inouye	Microseris lanceolata (Walp.) Sch.Bip.	Compositae	High	High	0,08	0,12	0,21	Prober SM, Spindler LH, Brown AHD (1998) Conservation of the Grassy White Box Woodlands : Effects of Remnant Population Size on Genetic Diversity in the Allotetraploid Herb Microseris lanceolata. Conserv Biol 12: 1279–1290.
Inouye	Brachyscome scapigera (Sieber ex Spreng.) DC.	Compositae	Low	High	0,07	0,13	0,22	Godfree R, Lepschi B, Mallinson D (2004) Ecological filtering of exotic plants in an Australian sub-alpine environment. J Veg Sci 15: 227–236.<br>Benson D, McDougall L (1994) Ecology of Sydney plant species part 2: dicotyledon families Asteraceae to Buddlejaceae. Cunninghamia 3: 789–1004.<br>Ali SI (1968) Senecio lautus complex in Australia IV. The biology of the complex. Phyton-Annales Rei Botanicae 13: 53–62.
Inouye	Leucopogon montanus (R.Br.) J.H.Willis	Ericaceae	High	High	0,07	0,19	0,33	http://avh.ala.org.au<br>http://encyclopaedia.alpinegardensociety.net
Inouye	Pentachondra pumila R. Br.	Ericaceae	High	High	0,05	0,03	0,07	Godley EJ (1966) Breeding systems in New Zealand plants. New Zeal J Bot 4: 249–254.
Inouye	Stylidium graminifolium Sw.	Stylidaceae	Inter	Low	0,04	0,09	0,17	Willis AJ, Ash JE (1990) The Breeding Systems of Stylidium graminifolium and *Stylidium productum* (Stylidiaceae). Austr J Bot 38: 217-227.<br>Mcintyre S, Lavorelt S, Tremont RM (2014) Plant life-history attributes : their relationship to in herbaceous disturbance response vegetation. 83: 31–44.
Inouye	Leptorhynchos squamatus (Labill.) Less.	Compositae	Inter	High	0,03	0,12	0,29	Flann C, Ladiges P, Walsh N (2002) Morphological variation in Leptorhynchos squamatus (Gnaphalieae: Asteraceae). Aust Syst Bot 15: 205–219.
Inouye	Asperula gunnii Hook.f.	Rubiaceae	High	High	0,02	0,08	0,20	Renner SS, Ricklefs RE (1995) Dioecy and Its Correlates in the Flowering Plants. Am J Bot 82: 596–606.<br>Benson D, McDougall L (1994) Ecology of Sydney plant species part 2: dicotyledon families Asteraceae to Buddlejaceae. Cunninghamia 3: 789–1004.

Inouye	Brachyscome stolonifera G.L.Davis	Compositae	Low	High	0,02	0,19	0,41	Noyes RD (2007) Apomixis in the Asteraceae : Diamonds in the Rough. Funct Plant Sci Biotechnol 1: 207–222.
Inouye	Epacris microphylla R.Br.	Ericaceae	Inter	Low	0,02	0,02	0,06	Celebrezze T (2002) Effects of European honeybees (Apis mellifera) on the pollination ecology of bird-and insect-adapted Australian plants. PhD Thesis. University of Wollongong. Available from http://ro.uow.edu.au/theses/1046.<br>Westoby M, Rice B, Howell J (1990) Seed size and plant growth form as factors in dispersal spectra. Ecology 71: 1307-1315.
Inouye	Epilobium gunnianum Hausskn.	Onagraceae	Inter	High	0,02	0,07	0,17	Raven PH (1979) A survey of reproductive biology in Onagraceae. New Zeal J Bot 17: 575–593.<br>Raven PH, Engelhorn T (1971) New taxa and new combinations in Australasian Epilobium (Onagraceae). New Zeal J Bot 9: 345–350.
Inouye	Aciphylla simplicifolia (F.Muell.) Benth.	Apiaceae	High	High	0,01	0,00	0,02	Pickering CM, Hill W (2002) Reproductive ecology and the effect of altitude on sex ratios in the dioecious herb *Aciphylla simplicifolia* (Apiaceae). Austr J Bot 50: 289–300.<br>Thorsen MJ, Dickinson KJM, Seddon PJ (2009) Seed dispersal systems in the New Zealand flora. Persp Plant Ecol Evol Syst 11: 285–309.
Inouye	Brachyscome spathulata Gaudich.	Compositae	Low	High	0,01	0,04	0,10	Noyes RD (2007) Apomixis in the Asteraceae : Diamonds in the Rough. Funct Plant Sci Biotechnol 1: 207–222.<br>Benson D, McDougall L (1994) Ecology of Sydney plant species part 2: dicotyledon families Asteraceae to Buddlejaceae. Cunninghamia 3: 789–1004.
Inouye	Brachyscome sp.	Compositae	Low	High	0,01	0,10	0,22	Noyes RD (2007) Apomixis in the Asteraceae : Diamonds in the Rough. Funct Plant Sci Biotechnol 1: 207–222.<br>Benson D, McDougall L (1994) Ecology of Sydney plant species part 2: dicotyledon families Asteraceae to Buddlejaceae. Cunninghamia 3: 789–1004.
KArroyoI	Phacelia secunda J.F.Gmel.	Boraginaceae	Inter	High	0,26	0,23	0,08	Medan D, Montaldo NH, Devoto M, Mantese A, Vasellati V, et al. (2002) Plant-pollinator relationships at two altitudes in the Andes of Mendoza, Argentina. Artic, Antart Alp Res 34: 233–241.<br>Castor C (2002) Patrones, procesos y mecanismos de dispersión secundaria en plantas andinas de Chile central. PhD Thesis. Facultad de Ciencias, Universidad de Chile. 172pp. Available from: http://www.tesis.uchile.cl/handle/2250/106689
KArroyoI	Chuquiraga oppositifolia D.Don	Compositae	High	High	0,11	0,12	0,10	Muñoz AA, Arroyo MTK (2006) Pollen Limitation in the Insect- and Spatial Success of Reproductive Variation Andes Shrub in the Chilean pollinated (Asteraceae) Chuquiraga oppositifolia. Arctic, Antarct Alp Res 38: 608–613.<br>García Berguesio N (2006) Análisis florístico comparativo de la vegetación alto-andina de la cordillera de la costa y de los andes de Chile central. PhD Thesis. Facultad de Ciencias Agronómicas, Escuela de Agronomía, Universidad de Chile. 68 pp. Available from: http://www.tesis.uchile.cl/handle/2250/101825
KArroyoI	Chaetanthera euphrasioides (DC.) F.Meigen	Compositae	Low	High	0,09	0,08	0,08	Arroyo MTK, Muñoz MS, Henríquez C, Till-bottraud I (1997) Original article Erratic pollination , high selfing levels and their correlates and consequences in an altitudinally widespread above-tree-line species in the high Andes of Chile.<br>García Berguesio N (2006) Análisis florístico comparativo de la vegetación alto-andina de la cordillera de la costa y de los andes de Chile central. PhD Thesis. Facultad de Ciencias Agronómicas, Escuela de Agronomía, Universidad de Chile. 68 pp. Available from: http://www.tesis.uchile.cl/handle/2250/101825
KArroyoI	Sisyrinchium arenarium Poepp.	Iridaceae	High	High	0,08	0,09	0,07	Arroyo M, Uslar P (1993) Breeding systems in a temperate mediterranean-type climate montane sclerophyllous forest in central Chile. Bot J Linn Soc 111: 83–102.<br>Castor C (2002) Patrones, procesos y mecanismos de dispersión secundaria en plantas andinas de Chile central. PhD Thesis. Facultad de Ciencias, Universidad de Chile. 172pp. Available from: http://www.tesis.uchile.cl/handle/2250/106689
KArroyoI	Mulinum spinosum Pers.	Apiaceae	High	High	0,08	0,14	0,14	Kalin Arroyo, M. T., & Uslar, P. (1993). Breeding systems in a temperate Mediterranean-type climate montane sclerophyllous forest in central Chile. Botanical journal of the Linnean Society, 111(1), 83-102.<br>Castor C (2002) Patrones, procesos y mecanismos de dispersión secundaria en plantas andinas de Chile central. PhD Thesis. Facultad de Ciencias, Universidad de Chile. 172pp. Available from: http://www.tesis.uchile.cl/handle/2250/106689
KArroyoI	Cerastium arvense L.	Caryophyllaceae	Inter	High	0,06	0,11	0,13	Quiroga MP, Premoli AC, Ezcurra C (2002) Morphological and isozyme variation in Cerastium arvense (Caryophyllaceae) in the southern Andes. Can J Bot 80: 786–795.<br>Castor C (2002) Patrones, procesos y mecanismos de dispersión secundaria en plantas andinas de Chile central. PhD Thesis. Facultad de Ciencias, Universidad de Chile. 172pp. Available from: http://www.tesis.uchile.cl/handle/2250/106689
KArroyoI	Alstroemeria pallida Graham	Alstroemeriaceae	Inter	Low	0,05	0,04	0,09	Arroyo M, Uslar P (1993) Breeding systems in a temperate mediterranean-type climate montane sclerophyllous forest in central Chile. Bot J Linn Soc 111: 83–102.<br>Cavieres, L. A., Papic, C., & Castor, C. (1999). Altitudinal variation in seed dispersal syndromes of the alpine vegetation of the rıo Molina basin, central Chile (33 S). Gayana Bot, 56, 115-123.
KArroyoI	Berberis empetrifolia Lam.	Berberidaceae	Inter	High	0,05	0,07	0,13	Kalin Arroyo MTK, Squeo F (2012) Relationships between plant breeding systems and pollination. In: Kawano S, editor. Biological Approaches and Evolutionary Trends in Plants. London: Elsevier. 430pp.<br>Cavieres, L. A., Papic, C., & Castor, C. (1999). Altitudinal variation in seed dispersal syndromes of the alpine vegetation of the rıo Molina basin, central Chile (33 S). Gayana Bot, 56, 115-123.
KArroyoI	Madia sativa Molina	Compositae	Low	High	0,05	0,08	0,10	Celedón-Neghme C, Gonzáles WL, Gianoli E (2006) Cost and benefits of attractive floral traits in the annual species Madia sativa (Asteraceae). Evol Ecol 21: 247–257.<br>Castor C (2002) Patrones, procesos y mecanismos de dispersión secundaria en plantas andinas de Chile central. PhD Thesis. Facultad de Ciencias, Universidad de Chile. 172pp. Available from: http://www.tesis.uchile.cl/handle/2250/106689
KArroyoI	Perezia carthamoides (D.Don) Hook. & Arn.	Compositae	Low	High	0,05	0,10	0,11	Muñoz A a., Cavieres L a. (2008) The presence of a showy invasive plant disrupts pollinator service and reproductive output in native alpine species only at high densities. J Ecol 96: 459–467.<br>García Berguesio N (2006) Análisis florístico comparativo de la vegetación alto-andina de la cordillera de la costa y de los andes de Chile central. PhD Thesis. Facultad de Ciencias Agronómicas, Escuela de Agronomía, Universidad de Chile. 68 pp. Available from: http://www.tesis.uchile.cl/handle/2250/101825
KArroyoI	Anarthrophyllum cumingii (Hook. & Arn.) F.Phil.	Leguminosae	High	Low	0,04	0,05	0,08	Rozzi R (1990) Períodos de floración y especies de polinizadores en poblaciones de *Anarthrophyllum cumingii* y *Chuquiraga oppositifolia* que crecen sobre laderas de exposición norte y sur. Master Thesis, Facultad de Ciencias, Universidad de Chile.<br>Cavieres, L. A., Papic, C., & Castor, C. (1999). Altitudinal variation in seed dispersal syndromes of the alpine vegetation of the rıo Molina basin, central Chile (33 S). Gayana Bot, 56, 115-123.
KArroyoI	Quinchamalium chilense Molina	Schoepfiaceae	Inter	Low	0,04	0,12	0,19	Riveros M, Arroyo MTK, Humaña AM (1987) An unusual kind of distyly in *Quinchamalium chilense* (Santalaceae) on Volcan Casablanca, Southern Chile.Am J Bot 74: 313-320.<br>García Berguesio N (2006) Análisis florístico comparativo de la vegetación alto-andina de la cordillera de la costa y de los andes de Chile central. PhD Thesis. Facultad de Ciencias Agronómicas, Escuela de Agronomía, Universidad de Chile. 68 pp. Available from: http://www.tesis.uchile.cl/handle/2250/101825
KArroyoI	Schizanthus hookeri Gillies ex Graham	Solanaceae	Low	Low	0,04	0,07	0,12	Pérez F, Arroyo MTK, Medel R, Hershkovitz MA (2006) Ancestral reconstruction of flower morphology and pollination systems in Schizanthus (Solanaceae). Am J Bot 93: 1029-1038.<br>García Berguesio N (2006) Análisis florístico comparativo de la vegetación alto-andina de la cordillera de la costa y de los andes de Chile central. PhD Thesis. Facultad de Ciencias Agronómicas, Escuela de Agronomía, Universidad de Chile. 68 pp. Available from: http://www.tesis.uchile.cl/handle/2250/101825
KArroyoI	Calceolaria mollissima Walp.	Calceolariaceae	Inter	Low	0,03	0,09	0,12	Sérsic A, *unpublished data*; Cosacov A, *unpublished data*
KArroyoI	Leucocoryne ixioides (Sims) Lindl.	Amaryllidaceae	High	Low	0,01	0,01	0,07	Arroyo M, Uslar P (1993) Breeding systems in a temperate mediterranean-type climate montane sclerophyllous forest in central Chile. Bot J Linn Soc 111: 83–102.<br>García Berguesio N (2006) Análisis florístico comparativo de la vegetación alto-andina de la cordillera de la costa y de los andes de Chile central. PhD Thesis. Facultad de Ciencias Agronómicas, Escuela de Agronomía, Universidad de Chile. 68 pp. Available from: http://www.tesis.uchile.cl/handle/2250/101825
KArroyoI	Rhodophiala montana (Phil.) Traub	Amaryllidaceae	Low	High	0,01	0,06	0,15	Ladd PG, Arroyo MTK (2009) Comparisons of breeding systems between two sympatric species, Nastanthus spathulatus (Calyceraceae) and Rhodophiala rhodolirion (Amaryllidaceae), in the high Andes of central Chile. Plant Species Biol 24: 2–10.<br>Cavieres, L. A., Papic, C., & Castor, C. (1999). Altitudinal variation in seed dispersal syndromes of the alpine vegetation of the rıo Molina basin, central Chile (33 S). Gayana Bot, 56, 115-123.
KArroyoI	Adesmia montana Phil.	Leguminosae	High	High	0,01	0,01	0,03	Arroyo M, Uslar P (1993) Breeding systems in a temperate mediterranean-type climate montane sclerophyllous forest in central Chile. Bot J Linn Soc 111: 83–102.<br>García Berguesio N (2006) Análisis florístico comparativo de la vegetación alto-andina de la cordillera de la costa y de los andes de Chile central. PhD Thesis. Facultad de Ciencias Agronómicas, Escuela de Agronomía, Universidad de Chile. 68 pp. Available from: http://www.tesis.uchile.cl/handle/2250/101825

KArroyoI	Lupinus microcarpus Sims	Leguminosae	Inter	Low	0,01	0,08	0,25	Drummond CS, Hamilton MB (2007) Hierarchical components of genetic variation at a species boundary: population structure in two sympatric varieties of Lupinus microcarpus (Leguminosae). Mol Ecol 16: 753–769. Available: http://www.ncbi.nlm.nih.gov/pubmed/17284209. Accessed 16 August 2014.<br>García Berguesio N (2006) Análisis florístico comparativo de la vegetación alto-andina de la cordillera de la costa y de los andes de Chile central. PhD Thesis. Facultad de Ciencias Agronómicas, Escuela de Agronomía, Universidad de Chile. 68 pp. Available from: http://www.tesis.uchile.cl/handle/2250/101825
KArroyoI	Malesherbia linearifolia (Cav.) Pers.	Passifloraceae	Low	Low	0,01	0,07	0,17	Arroyo M, Uslar P (1993) Breeding systems in a temperate mediterranean-type climate montane sclerophyllous forest in central Chile. Bot J Linn Soc 111: 83–102.<br>García Berguesio N (2006) Análisis florístico comparativo de la vegetación alto-andina de la cordillera de la costa y de los andes de Chile central. PhD Thesis. Facultad de Ciencias Agronómicas, Escuela de Agronomía, Universidad de Chile. 68 pp. Available from: http://www.tesis.uchile.cl/handle/2250/101825
KArroyoI	Clarkia tenella (Cav.) H.F.Lewis & M.R.Lewis	Onagraceae	Low	Low	0,01	0,08	0,25	Raven PH, Lewis H (1959) The relationship of clarkias from two continents. Brittonia 11: 193-205.<br>García Berguesio N (2006) Análisis florístico comparativo de la vegetación alto-andina de la cordillera de la costa y de los andes de Chile central. PhD Thesis. Facultad de Ciencias Agronómicas, Escuela de Agronomía, Universidad de Chile. 68 pp. Available from: http://www.tesis.uchile.cl/handle/2250/101825
KArroyoI	Acaena pinnatifida Ruiz & Pav.	Rosaceae	Low	High	0,01	0,17	0,37	Marticorena A (2006) Revisión del género Acaena (Rosaceae) en Chile. Ann Missouri Bot Gard 93: 412–454.<br>Bernardello G, Anderson GJ, Stuessy TF, Crawford DJ (2006) The angiosperm flora of the Archipelago Juan Fernandez (Chile): origin and dispersal. Can J Bot 84: 1266–1281.
KArroyoI	Calceolaria arachnoides Benth.	Calceolariaceae	High	Low	0,01	0,02	0,05	Sérsic A, *unpublished data*; Cosacov A, *unpublished data*
KArroyoI	Calceolaria biflora Lam.	Calceolariaceae	Low	Low	0,01	0,00	0,01	Sérsic A, *unpublished data*; Cosacov A, *unpublished data*
KArroyoI	Calceolaria sp.	Calceolariaceae	High	Low	0,01	0,08	0,25	Sérsic A, *unpublished data*; Cosacov A, *unpublished data*
KArroyoIII	Azorella madreporica Clos	Apiaceae	High	Low	0,10	0,26	0,16	Fajardo A, Quiroz CL, Cavieres L a. (2008) Distinguishing colonisation modes from spatial structures in populations of the cushion plant Azorella madreporica in the high-Andes of central Chile. Austral Ecol 33: 703–712.<br>Cavieres, L. A., Papic, C., & Castor, C. (1999). Altitudinal variation in seed dispersal syndromes of the alpine vegetation of the rıo Molina basin, central Chile (33 S). Gayana Bot, 56, 115-123.
KArroyoIII	Nassauvia lagascae Hauman	Compositae	High	High	0,06	0,24	0,24	López PG, Tremetsberger K, Stuessy TF, Gómez-González S, Jiménez A, et al. (2010) Patterns of genetic diversity in colonizing plant species: Nassauvia lagascae var. lanata (Asteraceae: Mutisieae) on Volcan Lonquimay, Chile. Am J Bot 97: 423–432.
KArroyoIII	Nototriche compacta (Gay) A.W. Hill	Malvaceae	Low	Low	0,06	0,24	0,24	García-Franco JG, Arroyo MTK (1995) Breeding System, Sex Ratio and Individual Size of the Gynodioecious Nototriche compacta (Malvaceae) in the Andes of Central Chile. Plant Species Biol 10: 147–153.<br>Cavieres, L. A., Papic, C., & Castor, C. (1999). Altitudinal variation in seed dispersal syndromes of the alpine vegetation of the rıo Molina basin, central Chile (33 S). Gayana Bot, 56, 115-123.
KArroyoIII	Caiophora coronata (Gillies ex Arnott) Hook. & Arn.	Loasaceae	Inter	Low	0,05	0,03	0,09	Cocucci A, Sersic A (1998) Evidence of rodent pollination in Cajophora coronata (Loasaceae). Plant Syst Evol 211: 113–128.<br>Ackermann M (2011) Studies on systematics, morphology and taxonomy of Caiophora and reproductive biology of Loasaceae and Mimulus (Phrymaceae). PhD Thesis. Fachbereich Biologie, Chemie, Pharmazie der Freien Universität Berlin
KArroyoIII	Chaetanthera apiculata F.Meigen	Compositae	Low	High	0,03	0,07	0,18	Torres-Díaz C, Cavieres LA, Muñoz-Ramírez C, Arroyo MTK (2007) Consecuencias de las variaciones microclimáticas sobre la visita de insectos polinizadores en dos especies de *Chaetanthera* (Asteraceae) en los Andes de Chile central. Rev chilena de hist nat 80: 455-468.
KArroyoIII	Chaetanthera lycopodioides (Remy) Cabrera ex Cabrera	Compositae	Low	High	0,03	0,07	0,18	Torres-Díaz C, Cavieres LA, Muñoz-Ramírez C, Arroyo MTK (2007) Consecuencias de las variaciones microclimáticas sobre la visita de insectos polinizadores en dos especies de *Chaetanthera* (Asteraceae) en los Andes de Chile central. Rev chilena de hist nat 80: 455-468.
KArroyoIII	Nastanthus scapigerus (J.Rémy) Miers	Calyceraceae	High	High	0,02	0,02	0,10	Ladd PG, Arroyo MTK (2009) Comparisons of breeding systems between two sympatric species, Nastanthus spathulatus (Calyceraceae) and Rhodophiala rhodolirion (Amaryllidaceae), in the high Andes of central Chile. Plant Species Biol 24: 2–10.<br>García Berguesio N (2006) Análisis florístico comparativo de la vegetación alto-andina de la cordillera de la costa y de los andes de Chile central. PhD Thesis. Facultad de Ciencias Agronómicas, Escuela de Agronomía, Universidad de Chile. 68 pp. Available from: http://www.tesis.uchile.cl/handle/2250/101825
KArroyoIII	Chaetanthera flabellata D.Don	Compositae	High	Low	0,02	0,04	0,10	Torres-Díaz C, Cavieres LA, Muñoz-Ramírez C, Arroyo MTK (2007) Consecuencias de las variaciones microclimáticas sobre la visita de insectos polinizadores en dos especies de Chaetanthera (Asteraceae) en los Andes de Chile central. Rev chilena de hist nat 80: 455-468.<br>Castor C (2002) Patrones, procesos y mecanismos de dispersión secundaria en plantas andinas de Chile central. PhD Thesis. Facultad de Ciencias, Universidad de Chile. 172pp. Available from: http://www.tesis.uchile.cl/handle/2250/106689
KArroyoIII	Ranunculus peduncularis Sm.	Ranunculaceae	Low	High	0,02	0,11	0,49	Arroyo MTK, von Bohlen CP, Cavieres L, Marticorena C (1992) Survey of the alpine flora of Torres del Paine National Park, Chile. Gayana Bot 49: 47-70.<br>San Martín C, Pérez Y, Montenegro D, Álvarez M (2011) Diversity, habit and habitat of aquatic vascular macrophytes of the western patagonia (Aisén region, Chile). Anales Instituto Patagonia (Chile) 39: 23-41.
Kevan	Dryas integrifolia Vahl	Rosaceae	Inter	High	0,61	0,38	0,10	Philipp M, Siegismund HR (2003) What can morphology and isozymes tell us about the history of the Dryas integrifolia-octopetala complex? Mol Ecol 12: 2231–2242.
Kevan	Salix arctica Pall.	Salicaceae	High	High	0,30	0,22	0,13	Kevan P (1972) Insect pollination of high arctic flowers. J Ecol 60: 831–847.<br>http://www.flora.dempstercountry.org
Kevan	Arnica angustifolia Vahl	Compositae	Low	High	0,28	0,19	0,14	Kevan P (1972) Insect pollination of high arctic flowers. J Ecol 60: 831–847.
Kevan	Saxifraga oppositifolia L.	Saxifragaceae	Low	High	0,27	0,21	0,13	Kevan P (1972) Insect pollination of high arctic flowers. J Ecol 60: 831–847.<br>http://www.nativeplantnetwork.org
Kevan	Potentilla nivea L.	Rosaceae	Low	Low	0,18	0,26	0,19	Kevan P (1972) Insect pollination of high arctic flowers. J Ecol 60: 831–847.<br>http://www.seed-dispersal.info
Kevan	Stellaria longipes Goldie	Caryophyllaceae	Inter	High	0,12	0,12	0,14	Philipp M (1980) Reproductive biology of Stellaria longipes Goldie as revealed by a cultivation experiment. New Phytol 85: 557–569.<br>Bartuszevige AM, Endress B (2008) Do ungulates facilitate native and exotic plant spread? J Arid Environ 72: 904–913.
Kevan	Papaver radicatum Rottb.	Papaveraceae	Low	Low	0,10	0,24	0,21	Kevan P (1972) Insect pollination of high arctic flowers. J Ecol 60: 831–847.<br>Bruun HH, Lundgren R, Philipp M (2008) Enhancement of local species richness in tundra by seed dispersal through guts of muskox and barnacle goose. Oecologia 155: 101–110.
Kevan	Pedicularis arctica R. Br.	Orobanchaceae	Low	Low	0,10	0,13	0,17	Kevan P (1972) Insect pollination of high arctic flowers. J Ecol 60: 831–847.

Kevan	Lesquerella arctica (Wormsk. ex Hornem.) S.Watson	Brassicaceae	Low	High	0,09	0,15	0,18	Kevan P (1972) Insect pollination of high arctic flowers. J Ecol 60: 831–847.<br>Payson EB (1921) A monograph of the genus Lesquerella. Ann Miss Bot Gard 8: 103-236.
Kevan	Taraxacum arctogenum Dahlst.	Compositae	Low	High	0,09	0,11	0,17	Kevan P (1972) Insect pollination of high arctic flowers. J Ecol 60: 831–847.
Kevan	Cerastium alpinum L.	Caryophyllaceae	Low	Low	0,03	0,14	0,28	Molau U (1993) Relationships between flowering phenology and Life History in Relationships Flowering Phenology Strategies in Tundra Plants. Arct Alp Res 25: 391–402. .<br>Welling P, Tolvanen A, Laine K (2012) Plant traits : Their role in the regeneration of alpine plant communities in sub-arctic Finland. J Veg Sci 16: 183–190.
Kevan	Saxifraga tricuspidata Rottb.	Saxifragaceae	Low	Low	0,03	0,11	0,23	Kevan P (1972) Insect pollination of high arctic flowers. J Ecol 60: 831–847.<br>Graae B, Pagh S, Bruun H (2004) An Experimental Evaluation of the Arctic Fox (Alopex lagopus) as a Seed Disperser. Arctic, Antarct Alp Res 36: 468–473.
Kevan	Erigeron compositus Pursh	Compositae	Low	High	0,03	0,06	0,16	Wiens D (1984) Ovule survivorship, brood size, life history, breeding systems, and reproductive success in plants. Oecologia 64: 47–53.<br>http://svalbardflora.net
Kevan	Epilobium latifolium L.	Onagraceae	Low	High	0,02	0,10	0,27	Kevan P (1972) Insect pollination of high arctic flowers. J Ecol 60: 831–847. .<br>Small E (1968) The systematics of autopolyploidy in Epilobium latifolium (Onagraceae). Brittonia 20: 169–181.
Kevan	Cassiope tetragona (L.) D.Don	Ericaceae	Low	Low	0,02	0,16	0,38	Fryxell P (1957) Mode of reproduction of higher plants. Bot Rev 23: 135–233.<br>Molau U (1997) Responses to natural climatic variation and experimental warming in two tundra plant species with contrasting life forms : Cassiope tetragona and Ranunculus nivalis. Glob Chang Biol 3: 97–107.
Kevan	Pedicularis capitata Adams	Orobanchaceae	Inter	Low	0,02	0,17	0,37	Kevan P (1972) Insect pollination of high arctic flowers. J Ecol 60: 831–847.
Kevan	Oxyria digyna (L.) Hill	Polygonaceae	Low	High	0,01	0,10	0,23	Molau U (1993) Relationships between flowering phenology and Life History in Relationships Flowering Phenology Strategies in Tundra Plants. Arct Alp Res 25: 391–402.<br>Welling P, Tolvanen A, Laine K (2012) Plant traits : Their role in the regeneration of alpine plant communities in sub-arctic Finland. J Veg Sci 16: 183–190.
Kevan	Silene taimyrensis (Tolm.) Bocquet	Caryophyllaceae	Low	High	0,01	0,10	0,23	Kevan P (1972) Insect pollination of high arctic flowers. J Ecol 60: 831–847. .<br>Nygren A (1951) Experimental studies in scandinavian alpine plants. Hereditas 37: 373-381.
Kevan	Erigeron eriocephalus J.Vahl	Compositae	Inter	High	0,01	0,10	0,27	http://svalbardflora.net
Medan I	Ochetophila nana (Clos) Kellermann, Medan & Aagesen	Rhamnaceae	High	Low	0,47	0,09	0,07	Medan D, Montaldo NH, Devoto M, Mantese A, Vasellati V, et al. (2002) Plant-pollinator relationships at two altitudes in the Andes of Mendoza, Argentina. Artic, Antart Alp Res 34: 233–241.<br>Cavieres, L. A., Papic, C., & Castor, C. (1999). Altitudinal variation in seed dispersal syndromes of the alpine vegetation of the rıo Molina basin, central Chile (33 S). Gayana Bot, 56, 115-123.
Medan I	Azorella monathos Clos	Apiaceae	High	Low	0,27	0,06	0,10	Medan D, Montaldo NH, Devoto M, Mantese A, Vasellati V, et al. (2002) Plant-pollinator relationships at two altitudes in the Andes of Mendoza, Argentina. Artic, Antart Alp Res 34: 233–241.<br>Cavieres, L. A., Papic, C., & Castor, C. (1999). Altitudinal variation in seed dispersal syndromes of the alpine vegetation of the rıo Molina basin, central Chile (33 S). Gayana Bot, 56, 115-123.
Medan I	Phacelia cf. secunda J.F.Gmel.	Boraginaceae	Inter	Low	0,13	0,07	0,15	Medan D, Montaldo NH, Devoto M, Mantese A, Vasellati V, et al. (2002) Plant-pollinator relationships at two altitudes in the Andes of Mendoza, Argentina. Artic, Antart Alp Res 34: 233–241.<br>Castor C (2002) Patrones, procesos y mecanismos de dispersión secundaria en plantas andinas de Chile central. PhD Thesis. Facultad de Ciencias, Universidad de Chile. 172pp. Available from: http://www.tesis.uchile.cl/handle/2250/106689
Medan I	Senecio looseri Cabrera	Compositae	Inter	High	0,13	0,12	0,17	Medan D, Montaldo NH, Devoto M, Mantese A, Vasellati V, et al. (2002) Plant-pollinator relationships at two altitudes in the Andes of Mendoza, Argentina. Artic, Antart Alp Res 34: 233–241.
Medan I	Olsynium junceum (E.Mey. ex C.Presl) Goldblatt	Iridaceae	Low	Low	0,13	0,11	0,14	Medan D, Montaldo NH, Devoto M, Mantese A, Vasellati V, et al. (2002) Plant-pollinator relationships at two altitudes in the Andes of Mendoza, Argentina. Artic, Antart Alp Res 34: 233–241.<br>García Berguesio N (2006) Análisis florístico comparativo de la vegetación alto-andina de la cordillera de la costa y de los andes de Chile central. PhD Thesis. Facultad de Ciencias Agronómicas, Escuela de Agronomía, Universidad de Chile. 68 pp. Available from: http://www.tesis.uchile.cl/handle/2250/101825
Medan I	Adesmia hemisphaerica Hauman	Leguminosae	Inter	Low	0,11	0,12	0,18	Medan D, Montaldo NH, Devoto M, Mantese A, Vasellati V, et al. (2002) Plant-pollinator relationships at two altitudes in the Andes of Mendoza, Argentina. Artic, Antart Alp Res 34: 233–241.<br>Cavieres, L. A., Papic, C., & Castor, C. (1999). Altitudinal variation in seed dispersal syndromes of the alpine vegetation of the rıo Molina basin, central Chile (33 S). Gayana Bot, 56, 115-123.
Medan I	Jaborosa laciniata (Miers) Hunz.	Solanaceae	Inter	High	0,11	0,03	0,10	Medan D, Montaldo NH, Devoto M, Mantese A, Vasellati V, et al. (2002) Plant-pollinator relationships at two altitudes in the Andes of Mendoza, Argentina. Artic, Antart Alp Res 34: 233–241.
Medan I	Astragalus nivicola Gomez-Sosa	Leguminosae	Inter	Low	0,07	0,12	0,24	Medan D, Montaldo NH, Devoto M, Mantese A, Vasellati V, et al. (2002) Plant-pollinator relationships at two altitudes in the Andes of Mendoza, Argentina. Artic, Antart Alp Res 34: 233–241.<br>Cavieres, L. A., Papic, C., & Castor, C. (1999). Altitudinal variation in seed dispersal syndromes of the alpine vegetation of the rıo Molina basin, central Chile (33 S). Gayana Bot, 56, 115-123.
Medan I	Montiopsis gilliesii (Hook. & Arn.) D.I. Ford	Montiaceae	Low	Low	0,04	0,05	0,19	Medan D, Montaldo NH, Devoto M, Mantese A, Vasellati V, et al. (2002) Plant-pollinator relationships at two altitudes in the Andes of Mendoza, Argentina. Artic, Antart Alp Res 34: 233–241.<br>Cavieres, L. A., Papic, C., & Castor, C. (1999). Altitudinal variation in seed dispersal syndromes of the alpine vegetation of the rıo Molina basin, central Chile (33 S). Gayana Bot, 56, 115-123.
Medan I	Oxalis erythrorrhiza Gillies ex Hook. & Arn.	Oxalidaceae	High	Low	0,04	0,05	0,14	Medan D, Montaldo NH, Devoto M, Mantese A, Vasellati V, et al. (2002) Plant-pollinator relationships at two altitudes in the Andes of Mendoza, Argentina. Artic, Antart Alp Res 34: 233–241.<br>Cavieres, L. A., Papic, C., & Castor, C. (1999). Altitudinal variation in seed dispersal syndromes of the alpine vegetation of the rıo Molina basin, central Chile (33 S). Gayana Bot, 56, 115-123.
Medan I	Perezia pilifera Hook. & Arn.	Compositae	Inter	High	0,04	0,05	0,19	Medan D, Montaldo NH, Devoto M, Mantese A, Vasellati V, et al. (2002) Plant-pollinator relationships at two altitudes in the Andes of Mendoza, Argentina. Artic, Antart Alp Res 34: 233–241.
Medan I	Senecio tricephalus Kuntze	Compositae	Inter	High	0,04	0,05	0,12	Medan D, Montaldo NH, Devoto M, Mantese A, Vasellati V, et al. (2002) Plant-pollinator relationships at two altitudes in the Andes of Mendoza, Argentina. Artic, Antart Alp Res 34: 233–241.

Medan I	Astragalus cruckshanksii (Hook. & Arn.) Griseb.	Leguminosae	Inter	Low	0,02	0,14	0,33	Medan D, Montaldo NH, Devoto M, Mantese A, Vasellati V, et al. (2002) Plant-pollinator relationships at two altitudes in the Andes of Mendoza, Argentina. Artic, Antart Alp Res 34: 233–241.<br>García Berguesio N (2006) Análisis florístico comparativo de la vegetación alto-andina de la cordillera de la costa y de los andes de Chile central. PhD Thesis. Facultad de Ciencias Agronómicas, Escuela de Agronomía, Universidad de Chile. 68 pp. Available from: http://www.tesis.uchile.cl/handle/2250/101825
Medan I	Calandrinia caespitosa Gillies ex Arn.	Portulacaceae	Low	Low	0,02	0,02	0,10	Medan D, Montaldo NH, Devoto M, Mantese A, Vasellati V, et al. (2002) Plant-pollinator relationships at two altitudes in the Andes of Mendoza, Argentina. Artic, Antart Alp Res 34: 233–241.<br>Cavieres, L. A., Papic, C., & Castor, C. (1999). Altitudinal variation in seed dispersal syndromes of the alpine vegetation of the rıo Molina basin, central Chile (33 S). Gayana Bot, 56, 115-123.
Medan I	Epilobium nivale Meyen	Onagraceae	Low	Low	0,02	0,07	0,19	Medan D, Montaldo NH, Devoto M, Mantese A, Vasellati V, et al. (2002) Plant-pollinator relationships at two altitudes in the Andes of Mendoza, Argentina. Artic, Antart Alp Res 34: 233–241.<br>García Berguesio N (2006) Análisis florístico comparativo de la vegetación alto-andina de la cordillera de la costa y de los andes de Chile central. PhD Thesis. Facultad de Ciencias Agronómicas, Escuela de Agronomía, Universidad de Chile. 68 pp. Available from: http://www.tesis.uchile.cl/handle/2250/101825
Medan I	Hypochaeris montana (Phil) Reiche	Compositae	Inter	High	0,02	0,02	0,10	Medan D, Montaldo NH, Devoto M, Mantese A, Vasellati V, et al. (2002) Plant-pollinator relationships at two altitudes in the Andes of Mendoza, Argentina. Artic, Antart Alp Res 34: 233–241.
Medan I	**Leucheria** candidissima Gillies & D.Don	Compositae	Inter	High	0,02	0,02	0,10	Medan D, Montaldo NH, Devoto M, Mantese A, Vasellati V, et al. (2002) Plant-pollinator relationships at two altitudes in the Andes of Mendoza, Argentina. Artic, Antart Alp Res 34: 233–241.
Medan I	Loasa incurva R.L.Pérez-Mor. & Crespo	Loasaceae	High	Low	0,02	0,05	0,14	Medan D, Montaldo NH, Devoto M, Mantese A, Vasellati V, et al. (2002) Plant-pollinator relationships at two altitudes in the Andes of Mendoza, Argentina. Artic, Antart Alp Res 34: 233–241.<br>García Berguesio N (2006) Análisis florístico comparativo de la vegetación alto-andina de la cordillera de la costa y de los andes de Chile central. PhD Thesis. Facultad de Ciencias Agronómicas, Escuela de Agronomía, Universidad de Chile. 68 pp. Available from: http://www.tesis.uchile.cl/handle/2250/101825
Medan I	Menonvillea hookeri Rollins	Brassicaceae	High	High	0,02	0,05	0,14	Medan D, Montaldo NH, Devoto M, Mantese A, Vasellati V, et al. (2002) Plant-pollinator relationships at two altitudes in the Andes of Mendoza, Argentina. Artic, Antart Alp Res 34: 233–241.<br>Cavieres, L. A., Papic, C., & Castor, C. (1999). Altitudinal variation in seed dispersal syndromes of the alpine vegetation of the rıo Molina basin, central Chile (33 S). Gayana Bot, 56, 115-123.
Medan II	Grindelia chiloensis (Cornel.) Cabrera	Compositae	High	High	0,47	0,20	0,10	Medan D, Montaldo NH, Devoto M, Mantese A, Vasellati V, et al. (2002) Plant-pollinator relationships at two altitudes in the Andes of Mendoza, Argentina. Artic, Antart Alp Res 34: 233–241.
Medan II	Ochetophila trinervis (Gillies ex Hook.) Poepp. ex Endl.	Rhamnaceae	High	Low	0,39	0,06	0,08	Medan D, Montaldo NH, Devoto M, Mantese A, Vasellati V, et al. (2002) Plant-pollinator relationships at two altitudes in the Andes of Mendoza, Argentina. Artic, Antart Alp Res 34: 233–241.<br>Medan D, Devoto M (2005) Reproductive ecology of a perennial outcrosser with a naturally dissected distribution. Plant Syst Evol 254: 173–184.
Medan II	Baccharis pingraea DC.	Compositae	High	High	0,15	0,02	0,10	Medan D, Montaldo NH, Devoto M, Mantese A, Vasellati V, et al. (2002) Plant-pollinator relationships at two altitudes in the Andes of Mendoza, Argentina. Artic, Antart Alp Res 34: 233–241.
Medan II	Larrea divaricata Cav.	Zygophyllaceae	Inter	High	0,08	0,05	0,12	Tadey M, Tadey JC, Tadey N (2009) Reproductive biology of five native plant species from the Monte Desert of Argentina. Bot J Linn Soc 161: 190–201.<br>Bonvissuto G, Busso C (2007) Seed rain in and between vegetation patches in arid Patagonia , Argentina. Phyt (Buenos Aires) 76: 47–59.
Medan II	Hysterionica jasionoides Willd.	Compositae	High	High	0,07	0,02	0,20	Medan D, Montaldo NH, Devoto M, Mantese A, Vasellati V, et al. (2002) Plant-pollinator relationships at two altitudes in the Andes of Mendoza, Argentina. Artic, Antart Alp Res 34: 233–241.
Medan II	Rosa rubiginosa L.	Rosaceae	High	High	0,07	0,00	0,14	Medan D, Montaldo NH, Devoto M, Mantese A, Vasellati V, et al. (2002) Plant-pollinator relationships at two altitudes in the Andes of Mendoza, Argentina. Artic, Antart Alp Res 34: 233–241.<br>http://www2.darwin.edu.ar/Proyectos/FloraArgentina/FA.asp
Medan II	Senecio subulatus (P.R.O.Bally) Jacobsen	Compositae	Inter	High	0,07	0,07	0,19	Medan D, Montaldo NH, Devoto M, Mantese A, Vasellati V, et al. (2002) Plant-pollinator relationships at two altitudes in the Andes of Mendoza, Argentina. Artic, Antart Alp Res 34: 233–241.
Medan II	Lathyrus sp.	Leguminosae	Inter	Low	0,04	0,08	0,13	Medan D, Montaldo NH, Devoto M, Mantese A, Vasellati V, et al. (2002) Plant-pollinator relationships at two altitudes in the Andes of Mendoza, Argentina. Artic, Antart Alp Res 34: 233–241.<br>García Berguesio N (2006) Análisis florístico comparativo de la vegetación alto-andina de la cordillera de la costa y de los andes de Chile central. PhD Thesis. Facultad de Ciencias Agronómicas, Escuela de Agronomía, Universidad de Chile. 68 pp. Available from: http://www.tesis.uchile.cl/handle/2250/101825
Medan II	Thelesperma megapotamicum (Spreng.) Kuntze	Compositae	High	High	0,04	0,08	0,22	Medan D, Montaldo NH, Devoto M, Mantese A, Vasellati V, et al. (2002) Plant-pollinator relationships at two altitudes in the Andes of Mendoza, Argentina. Artic, Antart Alp Res 34: 233–241.
Medan II	Arjona patagonica Hombr. & Jacquinot ex Decne.	Schoepfiaceae	High	High	0,03	0,01	0,11	Medan D, Montaldo NH, Devoto M, Mantese A, Vasellati V, et al. (2002) Plant-pollinator relationships at two altitudes in the Andes of Mendoza, Argentina. Artic, Antart Alp Res 34: 233–241.<br>Arroyo MTK, von Bohlen CP, Cavieres L, Marticorena C (1992) Survey of the alpine flora of Torres del Paine National Park, Chile. Gayana Bot 49: 47-70
Medan II	Junellia cf. toninii (Kuntze) Moldenke	Verbenaceae	Inter	High	0,03	0,01	0,11	Medan D, Montaldo NH, Devoto M, Mantese A, Vasellati V, et al. (2002) Plant-pollinator relationships at two altitudes in the Andes of Mendoza, Argentina. Artic, Antart Alp Res 34: 233–241.<br>Peralta PF, Múlgura ME (2011) El género Glandularia (Verbenaceae) en Argentina. Ann Missouri Bot Gard 98: 358–412.
Medan II	Oxalis sp.	Oxalidaceae	High	Low	0,03	0,07	0,13	Medan D, Montaldo NH, Devoto M, Mantese A, Vasellati V, et al. (2002) Plant-pollinator relationships at two altitudes in the Andes of Mendoza, Argentina. Artic, Antart Alp Res 34: 233–241.<br>García Berguesio N (2006) Análisis florístico comparativo de la vegetación alto-andina de la cordillera de la costa y de los andes de Chile central. PhD Thesis. Facultad de Ciencias Agronómicas, Escuela de Agronomía, Universidad de Chile. 68 pp. Available from: http://www.tesis.uchile.cl/handle/2250/101825
Medan II	Senecio filaginoides DC.	Compositae	High	High	0,03	0,09	0,22	Medan D, Montaldo NH, Devoto M, Mantese A, Vasellati V, et al. (2002) Plant-pollinator relationships at two altitudes in the Andes of Mendoza, Argentina. Artic, Antart Alp Res 34: 233–241.<br>http://www2.darwin.edu.ar/Proyectos/FloraArgentina/FA.asp
Medan II	Adesmia retrofracta Hook. & Arn.	Leguminosae	Inter	High	0,01	0,04	0,17	Medan D, Montaldo NH, Devoto M, Mantese A, Vasellati V, et al. (2002) Plant-pollinator relationships at two altitudes in the Andes of Mendoza, Argentina. Artic, Antart Alp Res 34: 233–241.<br>Ulibarri E, Burkart A (2000) Sinopsis de las especies de adesmia (leguminosae, adesmieae) de la Argentina. Darwiniana 38: 59–126.
Motten	Cardamine angustata O.E.Schulz	Brassicaceae	Low	Low	0,55	0,32	0,32	Motten A (1986) Pollination ecology of the spring wildflower community of a temperate deciduous forest. Ecol Monogr 56: 21–42.<br>Carlsen T, Bleeker W, Hurka H, Elven R, Brochmann C (2009) Biogeography and phylogeny of Cardamine (Brassicaceae). Ann Missouri Bot Gard 96: 215–236.
Motten	Stellaria pubera Michx.	Caryophyllaceae	Inter	High	0,48	0,21	0,31	Campbell D (1985) Pollinator sharing and seed set of Stellaria pubera : competition for pollination. Ecology 66: 544–553.<br>Williams S, Ward J (2006) Exotic seed dispersal by white-tailed deer in southern Connecticut. Nat Areas J 26: 383–390.

Motten	Erythronium umbilicatum C.R.Parks & Hardin	Liliaceae	High	Low	0,32	0,26	0,41	Motten A (1983) Reproduction of Erythronium umbilicatum (Liliaceae): pollination success and pollinator effectiveness. Oecologia 59: 351–359.
Motten	Hepatica nobilis var. obtusa (Pursh) Steyerm.	Ranunculaceae	Low	Low	0,27	0,27	0,43	Motten A (1982) Autogamy and Competition for Pollinators in *Hepatica americana* (Ranunculaceae). Am J Bot 69: 1296-1305.<br>Beattie A, Culver D (1981) The guild of myrmecochores in the herbaceous flora of west virginia forests. Ecology 62: 107–115.
Motten	Anemonella thalictroides (L.) Spach	Ranunculaceae	Low	Low	0,23	0,17	0,38	Motten A (1986) Pollination ecology of the spring wildflower community of a temperate deciduous forest. Ecol Monogr 56: 21–42.
Motten	Sanguinaria canadensis L.	Papaveraceae	Low	Low	0,16	0,22	0,45	Schemske DW, Willson MF, Melampy MN, Miller LJ, Verner L, et al. (1978) Flowering ecology of some spring woodland herbs. Ecology 59: 351–366.<br>Beattie A, Culver D (1981) The guild of myrmecochores in the herbaceous flora of west virginia forests. Ecology 62: 107–115.
Motten	Tiarella cordifolia L.	Saxifragaceae	High	Low	0,16	0,19	0,42	Motten A (1986) Pollination ecology of the spring wildflower community of a temperate deciduous forest. Ecol Monogr 56: 21–42.<br>Singleton R, Gardescu S, Marks PL, Geber MA (2001) Forest herb colonization of postagricultural forests in central New York State , USA: 325–338.
Motten	Viola papilionacea Pursh	Violaceae	Low	Low	0,11	0,11	0,29	Culver DC, Beattie a. J (1978) Myrmecochory in Viola: dynamics of seed-ant interactions in some West Virginia species. J Ecol 66: 53–72.
Motten	Uvularia sessilifolia L.	Colchicaceae	Inter	Low	0,11	0,15	0,37	Motten A (1986) Pollination ecology of the spring wildflower community of a temperate deciduous forest. Ecol Monogr 56: 21–42.
Motten	Trillium catesbaei Elliott	Melanthiaceae	Low	Low	0,07	0,22	0,51	Motten A (1986) Pollination ecology of the spring wildflower community of a temperate deciduous forest. Ecol Monogr 56: 21–42.<br>Zettler JA, Spira TP, Allen CR (2001) Yellow jackets (Vespula spp.) disperse Trillium (spp.) seeds in eastern North America. Am Midl Nat 146: 444–446.
Motten	Podophyllum peltatum L.	Berberidaceae	High	High	0,05	0,21	0,50	Swanson SD, Sohmer SH (1976) The biology of *Podophyllum peltatum* L. (Berberidaceae), the May Apple. II. The transfer of pollen and success of sexual reproduction. Bulletin Torr Bot Club 103: 223-226.<br>Rust RW, Roth RR (1981) Seed Production and Seedling Establishment in the Mayapple, *Podophyllum peltatum* L. Am Mid Nat 105: 51-60.
Ramirez	Hyptis dilatata Benth.	Lamiaceae	Low	High	0,19	0,07	0,09	Ramirez N, Brito Y (1990) Reproductive biology of a tropical palm swamp community in the venezuelan llanos. Am J Bot 77: 1260–1271.<br>Ramirez N, Brito Y (1988) Síndromes de dispersión de una comunidad de pantanos de palmeras (moricha!) en los Altos Llanos centrales venezolanos. Rev Chil Hist Nat 61: 53–60.
Ramirez	Cuphea odonellii Lourteig	Lythraceae	High	High	0,11	0,03	0,08	Ramirez N, Brito Y (1990) Reproductive biology of a tropical palm swamp community in the venezuelan llanos. Am J Bot 77: 1260–1271.<br>Ramirez N, Brito Y (1988) Síndromes de dispersión de una comunidad de pantanos de palmeras (moricha!) en los Altos Llanos centrales venezolanos. Rev Chil Hist Nat 61: 53–60.
Ramirez	Hyptis conferta Pohl ex Benth.	Lamiaceae	High	Low	0,09	0,05	0,11	Ramirez N, Brito Y (1990) Reproductive biology of a tropical palm swamp community in the venezuelan llanos. Am J Bot 77: 1260–1271.<br>Ramirez N, Brito Y (1988) Síndromes de dispersión de una comunidad de pantanos de palmeras (moricha!) en los Altos Llanos centrales venezolanos. Rev Chil Hist Nat 61: 53–60.
Ramirez	Pterolepis glomerata (Rottb.) Miq.	Melastomataceae	Low	High	0,09	0,05	0,11	Ramirez N, Brito Y (1990) Reproductive biology of a tropical palm swamp community in the venezuelan llanos. Am J Bot 77: 1260–1271.<br>Ramirez N, Brito Y (1988) Síndromes de dispersión de una comunidad de pantanos de palmeras (moricha!) en los Altos Llanos centrales venezolanos. Rev Chil Hist Nat 61: 53–60.
Ramirez	Syngonanthus caulescens (Poir.) Ruhland	Eriocaulaceae	Inter	High	0,08	0,06	0,16	Ramirez, N. & Brito, Y. 1990. Reproductive biology of a tropical palm swamp community in the Venezuelan llanos. *Am. J. Bot*. **77**, 1260-1271.<br>Gonçalves de Oliveira, P. 2009. Longevidade in situ e defesa química em sementes de Syngonanthus (Eriocaulaceae) dos campos rupestres de Minas Gerais, Brasil. PhD Thesis. Instituto de Ciencias Biológicas, Universidade Federal de Minas Gerais.
Ramirez	Aeschynomene pratensis Small	Leguminosae	Low	High	0,08	0,02	0,07	Ramirez N, Brito Y (1990) Reproductive biology of a tropical palm swamp community in the venezuelan llanos. Am J Bot 77: 1260–1271.<br>Ramirez N, Brito Y (1988) Síndromes de dispersión de una comunidad de pantanos de palmeras (moricha!) en los Altos Llanos centrales venezolanos. Rev Chil Hist Nat 61: 53–60.
Ramirez	Sauvagesia rubiginosa A.St.-Hil.	Ochnaceae	Inter	High	0,08	0,04	0,13	Ramirez N, Brito Y (1990) Reproductive biology of a tropical palm swamp community in the venezuelan llanos. Am J Bot 77: 1260–1271.<br>Ramirez N, Brito Y (1988) Síndromes de dispersión de una comunidad de pantanos de palmeras (moricha!) en los Altos Llanos centrales venezolanos. Rev Chil Hist Nat 61: 53–60.
Ramirez	Byttneria scabra L.	Malvaceae	High	High	0,08	0,03	0,12	Ramirez N, Brito Y (1990) Reproductive biology of a tropical palm swamp community in the venezuelan llanos. Am J Bot 77: 1260–1271.<br>Whitlock BA, Bayer C, Baum DA (2001) Phylogenetic relationships and floral evolution of the Byttnerioideae (Sterculiaceae or Malvaceae) based on sequences of the chloroplast gene, ndhF. Syst Bot 26: 420-437.
Ramirez	Xyris savanensis Miq.	Xyridaceae	High	High	0,08	0,09	0,21	Ramirez N, Brito Y (1990) Reproductive biology of a tropical palm swamp community in the venezuelan llanos. Am J Bot 77: 1260–1271.<br>Ramirez N, Brito Y (1988) Síndromes de dispersión de una comunidad de pantanos de palmeras (moricha!) en los Altos Llanos centrales venezolanos. Rev Chil Hist Nat 61: 53–60.
Ramirez	Caperonia palustris (L.) A.St.-Hil.	Euphorbiaceae	Inter	High	0,06	0,02	0,11	Ramirez N, Brito Y (1990) Reproductive biology of a tropical palm swamp community in the venezuelan llanos. Am J Bot 77: 1260–1271.<br>Ramirez N, Brito Y (1988) Síndromes de dispersión de una comunidad de pantanos de palmeras (moricha!) en los Altos Llanos centrales venezolanos. Rev Chil Hist Nat 61: 53–60.
Ramirez	Croton hirtus L'Hér.	Euphorbiaceae	Low	High	0,06	0,03	0,12	Ramirez N, Brito Y (1990) Reproductive biology of a tropical palm swamp community in the venezuelan llanos. Am J Bot 77: 1260–1271.<br>Ramirez N, Brito Y (1988) Síndromes de dispersión de una comunidad de pantanos de palmeras (moricha!) en los Altos Llanos centrales venezolanos. Rev Chil Hist Nat 61: 53–60.
Ramirez	Mimosa camporum Benth.	Leguminosae	Inter	High	0,06	0,06	0,18	Ramirez N, Brito Y (1990) Reproductive biology of a tropical palm swamp community in the venezuelan llanos. Am J Bot 77: 1260–1271.<br>Ramirez N, Brito Y (1988) Síndromes de dispersión de una comunidad de pantanos de palmeras (moricha!) en los Altos Llanos centrales venezolanos. Rev Chil Hist Nat 61: 53–60.
Ramirez	Desmodium barbatum (L.) Benth.	Leguminosae	Low	High	0,06	0,01	0,08	Ramirez N, Brito Y (1990) Reproductive biology of a tropical palm swamp community in the venezuelan llanos. Am J Bot 77: 1260–1271.<br>Moura FDBP, Duarte JMM, Lemos RPDL (2011) Floristic composition and dispersal syndromes at an urban remnant from the Atlantic forest in Brazilian Northeast. Acta Sci Biol Sci 33: 471–478.
Ramirez	Ludwigia decurrens Walter	Onagraceae	Low	High	0,06	0,02	0,08	Vieira AOS (2002) Biologia reprodutiva e hibridação em espécies sintópicas de *Ludwigia* (Onagraceae) no Sudeste do Brasil. PhD Thesis. Instituto de Biologia, Universidade de Campinas.
Ramirez	Melochia villosa (Mill.) Fawc. & Rendle	Malvaceae	High	Low	0,06	0,07	0,15	Ramirez N, Brito Y (1990) Reproductive biology of a tropical palm swamp community in the venezuelan llanos. Am J Bot 77: 1260–1271.<br>Cortés-Pérez F, León-Sicard TE (2003) Modelo conceptual del papel ecológico de la Hormiga Arriera *Atta laevigata* en los ecosistemas de sabana estacional (Vichada, Colombia). Caldasia 25: 403-417.
Ramirez	Clidemia capitellata (Bonpl.) D. Don	Melastomataceae	Low	High	0,04	0,00	0,04	Ramirez N, Brito Y (1990) Reproductive biology of a tropical palm swamp community in the venezuelan llanos. Am J Bot 77: 1260–1271.<br>Ramirez N, Brito Y (1988) Síndromes de dispersión de una comunidad de pantanos de palmeras (moricha!) en los Altos Llanos centrales venezolanos. Rev Chil Hist Nat 61: 53–60.
Ramirez	Miconia stephananthera Ule	Melastomataceae	Low	High	0,04	0,03	0,09	Ramirez N, Brito Y (1990) Reproductive biology of a tropical palm swamp community in the venezuelan llanos. Am J Bot 77: 1260–1271.<br>Ramirez N, Brito Y (1988) Síndromes de dispersión de una comunidad de pantanos de palmeras (moricha!) en los Altos Llanos centrales venezolanos. Rev Chil Hist Nat 61: 53–60.
Ramirez	Heliconia psittacorum L.f.	Heliconiaceae	Inter	High	0,04	0,00	0,07	Ramirez N, Brito Y (1990) Reproductive biology of a tropical palm swamp community in the venezuelan llanos. Am J Bot 77: 1260–1271.<br>Ramirez N, Brito Y (1988) Síndromes de dispersión de una comunidad de pantanos de palmeras (moricha!) en los Altos Llanos centrales venezolanos. Rev Chil Hist Nat 61: 53–60.

Ramirez	Spermacoce multiflora (DC.) Delprete	Rubiaceae	Low	High	0,04	0,09	0,21	Ramirez N, Brito Y (1990) Reproductive biology of a tropical palm swamp community in the venezuelan llanos. Am J Bot 77: 1260–1271.<br>Ramirez N, Brito Y (1988) Síndromes de dispersión de una comunidad de pantanos de palmeras (moricha!) en los Altos Llanos centrales venezolanos. Rev Chil Hist Nat 61: 53–60.
Ramirez	Xyris laxifolia Mart.	Xyridaceae	Low	High	0,04	0,10	0,23	Ramirez N, Brito Y (1990) Reproductive biology of a tropical palm swamp community in the venezuelan llanos. Am J Bot 77: 1260–1271.<br>Ramirez N, Brito Y (1988) Síndromes de dispersión de una comunidad de pantanos de palmeras (moricha!) en los Altos Llanos centrales venezolanos. Rev Chil Hist Nat 61: 53–60.
Ramirez	Montrichardia arborescens (L.) Schott	Araceae	Low	High	0,02	0,00	0,04	Gibernau M, Barabé D, Labat D, Cerdan P, Dejean A (2003) Reproductive biology of Montrichardia arborescens (Araceae) in French Guiana. J Trop Ecol 19: 103–107.<br>Lucas CM (2008) Within flood season variation in fruit consumption and seed dispersal by two characin fishes of the Amazon. Biotropica 40: 581-589.
Ramirez	Thalia geniculata L.	Marantaceae	Low	High	0,02	0,00	0,04	Ley AC, Claßen-bockhoff R (2012) Floral synorganization and its influence on mechanical isolation and autogamy in Marantaceae. Bot J Linn Soc 168: 300–322.<br>Gonçalves de Sousa D (2007) Dinamica de regeneraçao natural da espécie *Monotagma densiflorum* (Koern.) K. Schum. (Cantan), em floresta manejada de terra firme na regiao de moju-pa. Master thesis, Universidade Federal Rural da Amazônia.
Schemske	Claytonia virginica L.	Montiaceae	Inter	Low	0,69	0,38	0,32	Schemske, D. W., Willson, M. F., Melampy, M. N., Miller, L. J., Verner, L., Schemske, K. M. & Best, L. B. 1978. Flowering ecology of some spring woodland herbs. *Ecology* **59**, 351-366.<br>Beattie, A. J. & Culver, D. C. 1981. The guild of myrmecochores in the herbaceous flora of West Virginia forests. *Ecology* **62**, 107-115.
Schemske	Enemion biternatum (Torr. & A.Gray) Raf.	Ranunculaceae	Inter	Low	0,59	0,21	0,31	Schemske, D. W., Willson, M. F., Melampy, M. N., Miller, L. J., Verner, L., Schemske, K. M. & Best, L. B. 1978. Flowering ecology of some spring woodland herbs. *Ecology* **59**, 351-366.
Schemske	Cardamine concatenata (Michx.) O.Schwarz	Brassicaceae	Inter	Low	0,31	0,28	0,41	Schemske DW, Willson MF, Melampy MN, Miller LJ, Verner L, et al. (1978) Flowering ecology of some spring woodland herbs. Ecology 59: 351–366.<br>Beattie A, Culver D (1981) The guild of myrmecochores in the herbaceous flora of west virginia forests. Ecology 62: 107–115.
Schemske	Erythronium albidum Nutt.	Liliaceae	Inter	Low	0,16	0,14	0,43	Schemske DW, Willson MF, Melampy MN, Miller LJ, Verner L, et al. (1978) Flowering ecology of some spring woodland herbs. Ecology 59: 351–366.
Schemske	Dicentra canadensis (Goldie) Walp.	Papaveraceae	High	Low	0,03	0,14	0,57	Schemske DW, Willson MF, Melampy MN, Miller LJ, Verner L, et al. (1978) Flowering ecology of some spring woodland herbs. Ecology 59: 351–366.
Schemske	Dicentra cucullaria (L.) Bernh.	Papaveraceae	High	Low	0,03	0,14	0,57	Schemske DW, Willson MF, Melampy MN, Miller LJ, Verner L, et al. (1978) Flowering ecology of some spring woodland herbs. Ecology 59: 351–366.
Schemske	Sanguinaria canadensis L.	Papaveraceae	Low	Low	0,03	0,14	0,43	Schemske DW, Willson MF, Melampy MN, Miller LJ, Verner L, et al. (1978) Flowering ecology of some spring woodland herbs. Ecology 59: 351–366.
Vázquez	Alstroemeria aurea Graham	Alstroemeriaceae	Inter	Low	0,57	0,29	0,16	Vázquez DP, Simberloff D (2004) Indirect effects of an introduced ungulate on pollination and plant reproduction. Ecol Monogr 74: 281-308.<br>Souto C, Aizen M, Premoli A (2002) Effects of crossing distance and genetic relatedness on pollen performance in Alstroemeria aurea (Alstroemeriaceae). Am J … 89: 427–432.
Vázquez	Schinus patagonicus (Phil.) I.M. Johnst.	Anacardiaceae	High	High	0,30	0,20	0,18	Vázquez DP, Simberloff D (2004) Indirect effects of an introduced ungulate on pollination and plant reproduction. Ecol Monogr 74: 281-308.<br>Cavallero L, Aizen M a., Raffaele E (2012) Endozoochory decreases environmental filtering imposed to seedlings. J Veg Sci 23: 677–689.
Vázquez	Rosa rubiginosa L.	Rosaceae	Inter	High	0,24	0,18	0,22	Vázquez DP, Simberloff D (2004) Indirect effects of an introduced ungulate on pollination and plant reproduction. Ecol Monogr 74: 281-308.<br>Cavallero L, Aizen M a., Raffaele E (2012) Endozoochory decreases environmental filtering imposed to seedlings. J Veg Sci 23: 677–689.
Vázquez	Mutisia decurrens Cav.	Compositae	High	High	0,13	0,13	0,23	Medan D, Montaldo NH, Devoto M, Mantese A, Vasellati V, et al. (2002) Plant-pollinator relationships at two altitudes in the Andes of Mendoza, Argentina. Artic, Antart Alp Res 34: 233–241.
Vázquez	Berberis darwinii Hook.	Berberidaceae	High	High	0,12	0,21	0,32	Vázquez DP, Simberloff D (2004) Indirect effects of an introduced ungulate on pollination and plant reproduction. Ecol Monogr 74: 281-308.<br>Cavallero L, Aizen M a., Raffaele E (2012) Endozoochory decreases environmental filtering imposed to seedlings. J Veg Sci 23: 677–689.
Vázquez	Aristotelia chilensis (Molina) Stuntz	Elaeocarpaceae	High	High	0,07	0,07	0,23	Vázquez DP, Simberloff D (2004) Indirect effects of an introduced ungulate on pollination and plant reproduction. Ecol Monogr 74: 281-308.<br>Cavallero L, Aizen M a., Raffaele E (2012) Endozoochory decreases environmental filtering imposed to seedlings. J Veg Sci 23: 677–689.
Vázquez	Digitalis purpurea L.	Plantaginaceae	Low	Low	0,07	0,15	0,33	Grindeland JM (2008) Inbreeding depression and outbreeding depression in Digitalis purpurea: optimal outcrossing distance in a tetraploid. J Evol Biol 21: 716–726.
Vázquez	Berberis microphylla G.Forst.	Berberidaceae	High	High	0,04	0,20	0,45	Vázquez DP, Simberloff D (2004) Indirect effects of an introduced ungulate on pollination and plant reproduction. Ecol Monogr 74: 281-308.<br>Cavallero L, Aizen M a., Raffaele E (2012) Endozoochory decreases environmental filtering imposed to seedlings. J Veg Sci 23: 677–689.
Vázquez	Calceolaria crenatiflora Cav.	Calceolariaceae	Inter	Low	0,03	0,11	0,33	Vázquez DP, Simberloff D (2004) Indirect effects of an introduced ungulate on pollination and plant reproduction. Ecol Monogr 74: 281-308.
Vázquez	Tristerix corymbosus (L.) Kuijt	Loranthaceae	Low	High	0,03	0,15	0,43	Aizen MA (2005) Breeding system of Tristerix corymbosus (Loranthaceae), a winter-flowering mistletoe from the southern Andes. Aust J Bot 53: 357–361.<br>Amico GC, Rodríguez-Cabal M a., Aizen M (2009) The potential key seed-dispersing role of the arboreal marsupial Dromiciops gliroides. Acta Oecologica 35: 8–13.
