# Supplementary material for: The Robustness of Plant-Pollinator Assemblages: Linking Plant Interaction Patterns and Sensitivity to Pollinator Loss
Source: PLoS One. 2015 Feb 3;10(2):e0117243. doi: 10.1371/journal.pone.0117243 (PMC4315602; doi:10.1371/journal.pone.0117243)
Supplement: S3 Table — Scenarios linking plant probability of extinction to different plant traits associated with plant sensitivity to pollinator loss (i.e. plant generalization, dependence on pollinators, dispersal ability) or combinations of these traits. The random scenario (no trait is considered) was also explored. (PDF) [file pone.0117243.s003.pdf]

Scenario	Trait	Species probability of extinction
Random	None	$p_{ep} = \frac{1}{S_p}$
Plant generalization	Plant generalization on pollinators	$k_p \uparrow, p_{ep} \downarrow$
Plant dependence	Plant reproductive dependence on pollinators	$p_{epstrgdp} > p_{epintdp} > p_{epslidp}$
Plant dispersal	Plant dispersal ability	$p_{ephighda} > p_{eplowda}$
Plant dependence and dispersal	Plant reproductive dependence on pollinators and dispersal ability	$p_{ep} = p_{epdp} \cdot p_{epda}$
Plant generalization and dependence	Plant generalization on pollinators and plant reproductive dependence on pollinators	$p_{ep} = p_{epkp} \cdot p_{epdp}$
Plant generalization and dispersal	Plant generalization on pollinators and plant dispersal ability	$p_{ep} = p_{epkp} \cdot p_{epda}$
Plant generalization, dependence and dispersal	Plant generalization on pollinators, plant reproductive dependence on pollinators and plant dispersal ability	$p_{ep} = p_{epkp} \cdot p_{epdp} \cdot p_{epda}$

References: k_p = plant degree; p_{ep} = plant's probability of extinction; p_{epda} = plant's probability of extinction associated to dispersal ability; p_{epdp} = plant's probability of extinction associated to dependence on pollinators; p_{epkp} = plant's probability of extinction associated to generalization on pollinators; $p_{epstrdp}$ = plant's probability of extinction associated to strong dependence on pollinators; $p_{epintdp}$ = plant's probability of extinction associated to intermediate dependence on pollinators; $p_{epslida}$ = plant's probability of extinction associated to slight dependence on pollinators; $p_{ephighda}$ = plant's probability of extinction associated to high dispersal ability; $p_{eplowdp}$ = plant's probability of extinction associated to low dependence on pollinators.
