# Supplementary material for: The Robustness of Plant-Pollinator Assemblages: Linking Plant Interaction Patterns and Sensitivity to Pollinator Loss
Source: PLoS One. 2015 Feb 3;10(2):e0117243. doi: 10.1371/journal.pone.0117243 (PMC4315602; doi:10.1371/journal.pone.0117243)
Supplement: S4 Table — Sum of squares (SS), degrees of freedom (df), mean squares (MS), and F-ratio are shown for plant generalization on pollinators, contribution to nestedness and mean pollinator generalization. Probability values (p) obtained from permutation analyses are also shown. (PDF) [file pone.0117243.s004.pdf]

Response variable	Source	SS	df	MS	F-ratio	*p*
Plant generalization on pollinators	Dependence on pollinators	0.01	1	0.01	0.68	0.4
	Dispersal ability	0.03	1	0.03	1.85	0.18
	Dependence on pollinators x dispersal ability	0.03	1	0.03	1.45	0.24
	Residuals	3.24	188	0.017		
	Total	3.32	191			
Plant contribution to nestedness	Dependence on pollinators	0.01	1	0.01	1.78	0.19
	Dispersal ability	0.05	1	0.05	7.62	0.006
	Dependence on pollinators x dispersal ability	0.04	1	0.04	7.19	0.006
	Residuals	1.14	188	0.006		
	Total	1.24	191			
Mean pollinator generalization	Dependence on pollinators	0.05	1	0.05	3.37	0.07
	Dispersal ability	0.15	1	0.15	11.26	0.0013

Dependence on pollinators x dispersal ability	0.03	1	0.03	1.97	0.16
Residuals	2.54	188	0.014		
Total	2.76	191			
