# Supplementary material for: The Robustness of Plant-Pollinator Assemblages: Linking Plant Interaction Patterns and Sensitivity to Pollinator Loss
Source: PLoS One. 2015 Feb 3;10(2):e0117243. doi: 10.1371/journal.pone.0117243 (PMC4315602; doi:10.1371/journal.pone.0117243)
Supplement: S9 Table — Mean differences of robustness (Mean) between pairs of scenarios, and minimum (CI min) and maximum (CI max) limits of the 95% confidence interval of each difference are shown for the Medán II network. Pairwise comparisons were planned a-priori. Scenarios: dependence on pollinators (DP), dispersal ability (DA), dependence on pollinators and dispersal ability (DPDA), random (R), dependence on pollinators and generalization (DPG), dispersal ability and generalization (DAG), dependence on pollinators, dispersal ability and generalization (DPDAG) and generalization (G). (PDF) [file pone.0117243.s009.pdf]

| Extinction Scenario | DP | | | DA | | | DPDA | | | R | | | DPG | | | DAG | | | DPDAG | | | G | | |
|---|---|---|---|---|---|---|---|---|---|---|---|---|---|---|---|---|---|---|---|---|---|---|---|---|
| | Mean | CI min | CI max | Mean | CI min | CI max | Mean | CI min | CI max | Mean | CI min | CI max | Mean | CI min | CI max | Mean | CI min | CI max | Mean | CI min | CI max | Mean | CI min | CI max |
| DP | | | | | | | | | | 0.056 | 0.013 | 0.099 | 0.105 | 0.063 | 0.148 | | | | | | | 0.156 | 0.114 | 0.198 |
| DA | | | | | | | | | | 0.013 | -0.03 | 0.056 | | | | 0.095 | 0.053 | 0.137 | | | | 0.113 | 0.071 | 0.154 |
| DPDA | | | | | | | | | | 0.067 | 0.024 | 0.111 | | | | | | | 0.101 | 0.058 | 0.145 | 0.167 | 0.125 | 0.209 |
| R | 0.056 | 0.013 | 0.099 | 0.013 | -0.03 | 0.056 | 0.067 | 0.024 | 0.111 | | | | 0.049 | 0.007 | 0.092 | 0.082 | 0.04 | 0.124 | 0.034 | -0.008 | 0.077 | 0.099 | 0.058 | 0.141 |
| DPG | 0.105 | 0.063 | 0.148 | | | | | | | 0.049 | 0.007 | 0.092 | | | | | | | | | | 0.051 | 0.009 | 0.092 |
| DAG | | | | 0.095 | 0.053 | 0.137 | | | | 0.082 | 0.04 | 0.124 | | | | | | | | | | 0.018 | -0.023 | 0.058 |
| DPDAG | | | | | | | 0.101 | 0.058 | 0.145 | 0.034 | -0.008 | 0.077 | | | | | | | | | | 0.066 | 0.024 | 0.107 |
| G | 0.156 | 0.114 | 0.198 | 0.113 | 0.071 | 0.154 | 0.167 | 0.125 | 0.209 | 0.099 | 0.058 | 0.141 | 0.051 | 0.009 | 0.092 | 0.018 | -0.023 | 0.058 | 0.066 | 0.024 | 0.107 | | | |
