# Supplementary figures and images for: The Robustness of Plant-Pollinator Assemblages: Linking Plant Interaction Patterns and Sensitivity to Pollinator Loss
Source: PLoS One. 2015 Feb 3;10(2):e0117243. doi: 10.1371/journal.pone.0117243 (PMC4315602; doi:10.1371/journal.pone.0117243)

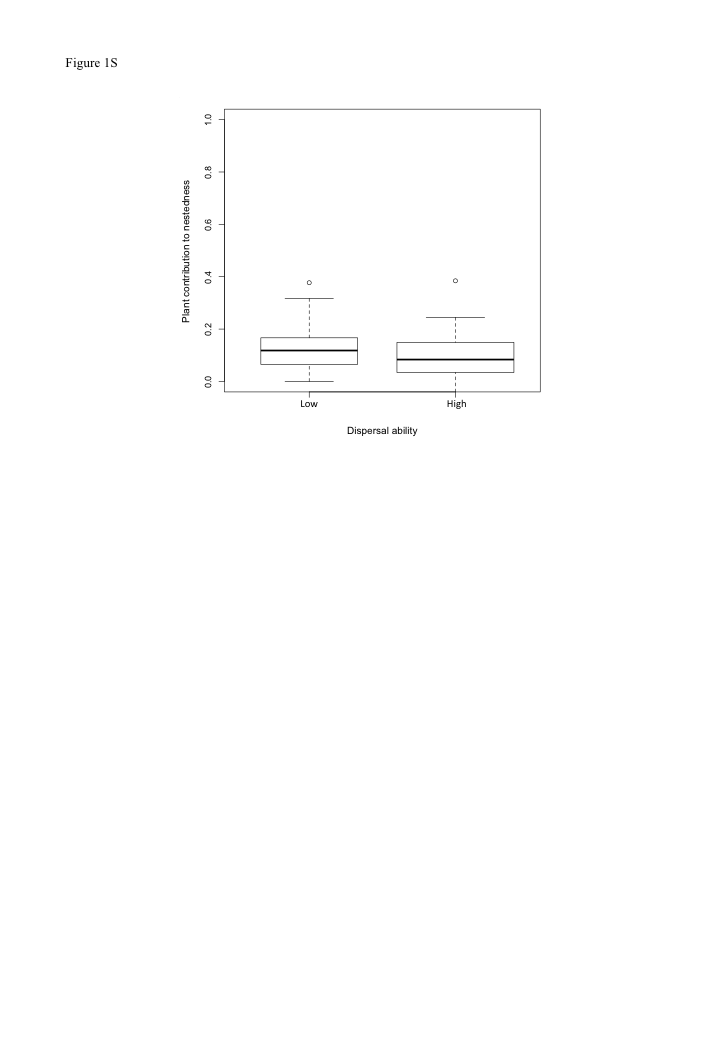

Supplement: S1 Fig — Box-plots of plant contribution to nestedness of species with different dispersal ability. Black lines within boxes represent median values. Upper and lower limits of boxes represent 1st and 3rd quartiles, respectively. Boxes were drawn with widths proportional to the number of observations in each group. “Low” and “High” refer to low and high-dispersal plants, respectively. (TIFF) [file pone.0117243.s015.tiff]
